# Supplementary figures and images for: Cross-Laboratory Analysis of Brain Cell Type Transcriptomes with Applications to Interpretation of Bulk Tissue Data
Source: eNeuro. 2017 Nov 30;4(6):ENEURO.0212-17.2017. doi: 10.1523/ENEURO.0212-17.2017 (PMC5707795; doi:10.1523/ENEURO.0212-17.2017)

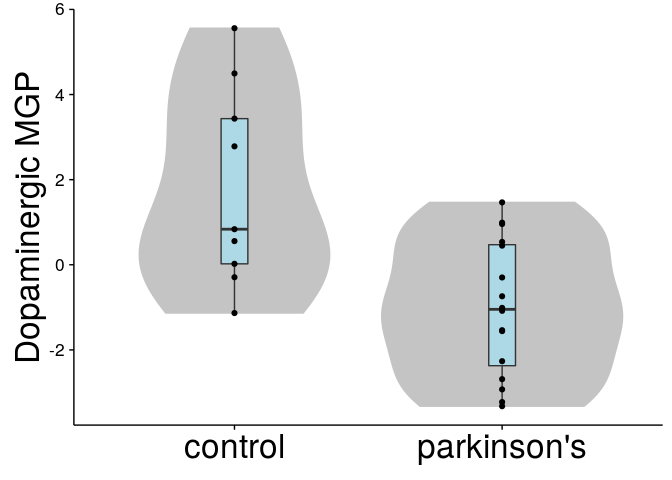

Supplement: Extended Data 1 [file enu006172455so3.zip › markerGeneProfile-master/README_files/figure-markdown_github-ascii_identifiers/unnamed-chunk-16-1.png]

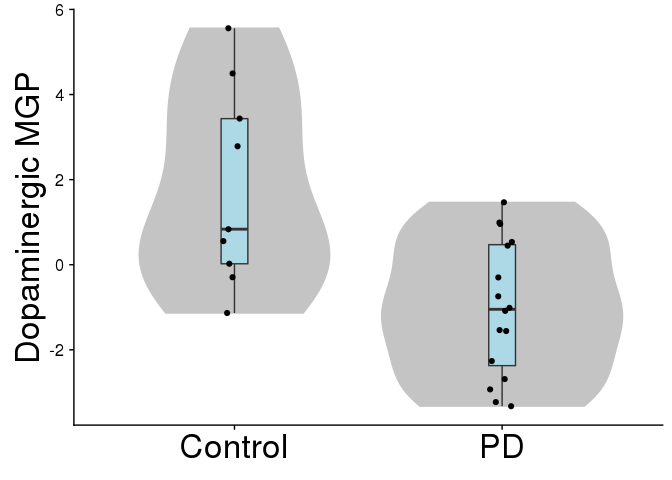

Supplement: Extended Data 1 [file enu006172455so3.zip › markerGeneProfile-master/README_files/figure-markdown_github-ascii_identifiers/unnamed-chunk-17-1.png]

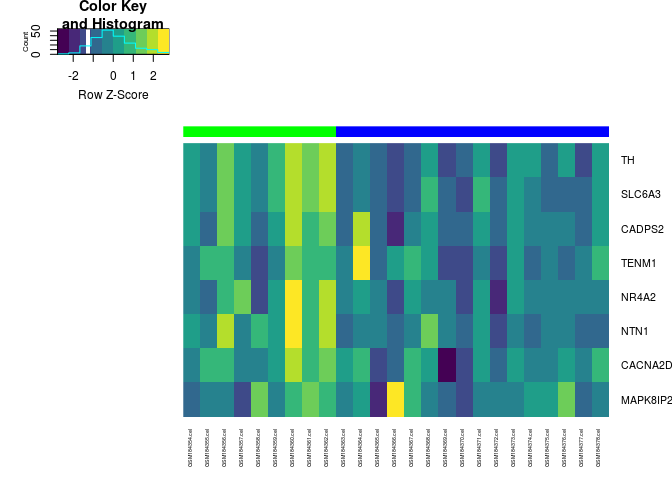

Supplement: Extended Data 1 [file enu006172455so3.zip › markerGeneProfile-master/README_files/figure-markdown_github-ascii_identifiers/unnamed-chunk-18-1.png]

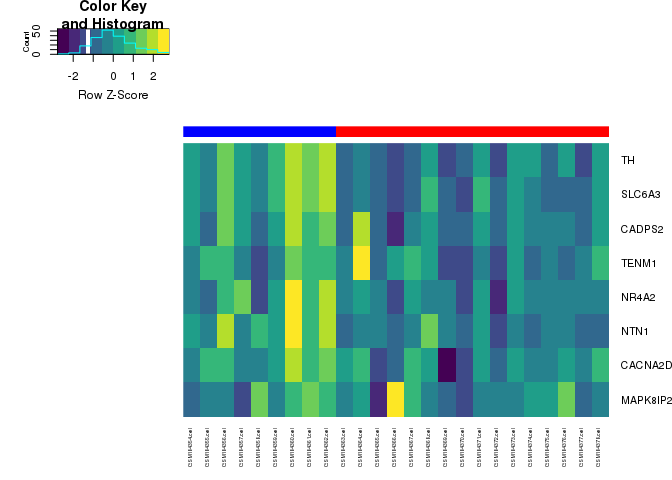

Supplement: Extended Data 1 [file enu006172455so3.zip › markerGeneProfile-master/README_files/figure-markdown_github-ascii_identifiers/unnamed-chunk-19-1.png]

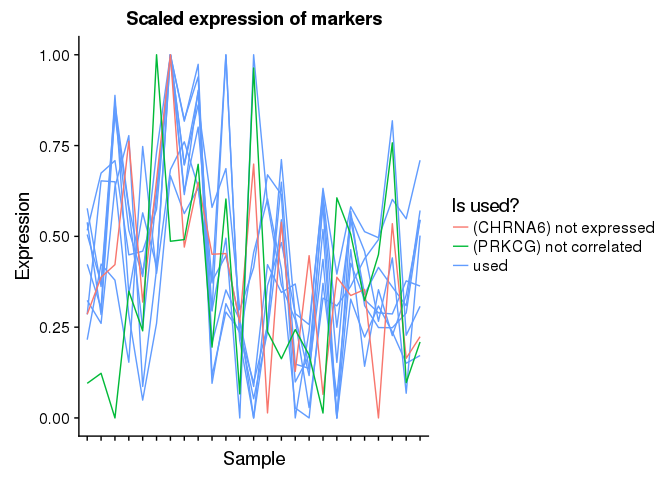

Supplement: Extended Data 1 [file enu006172455so3.zip › markerGeneProfile-master/README_files/figure-markdown_github-ascii_identifiers/unnamed-chunk-23-1.png]

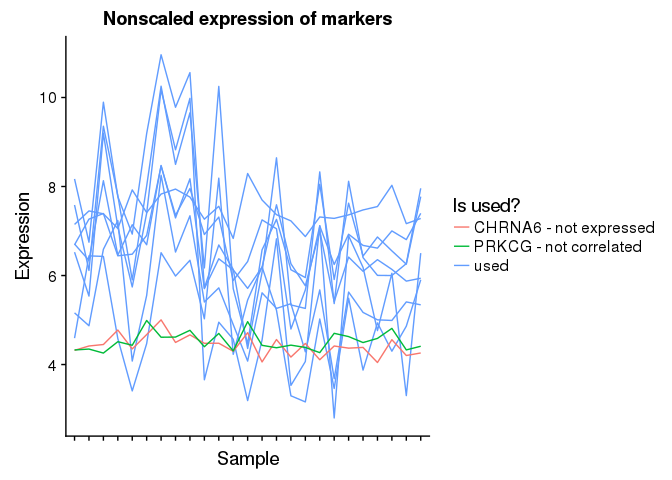

Supplement: Extended Data 1 [file enu006172455so3.zip › markerGeneProfile-master/README_files/figure-markdown_github-ascii_identifiers/unnamed-chunk-23-2.png]

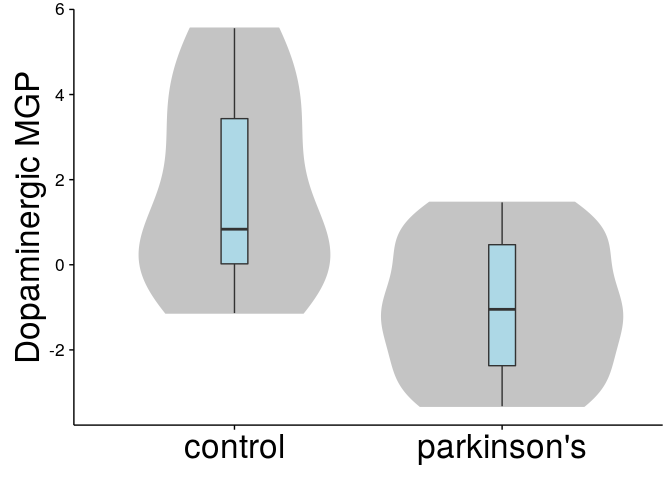

Supplement: Extended Data 1 [file enu006172455so3.zip › markerGeneProfile-master/README_files/figure-markdown_github/unnamed-chunk-16-1.png]

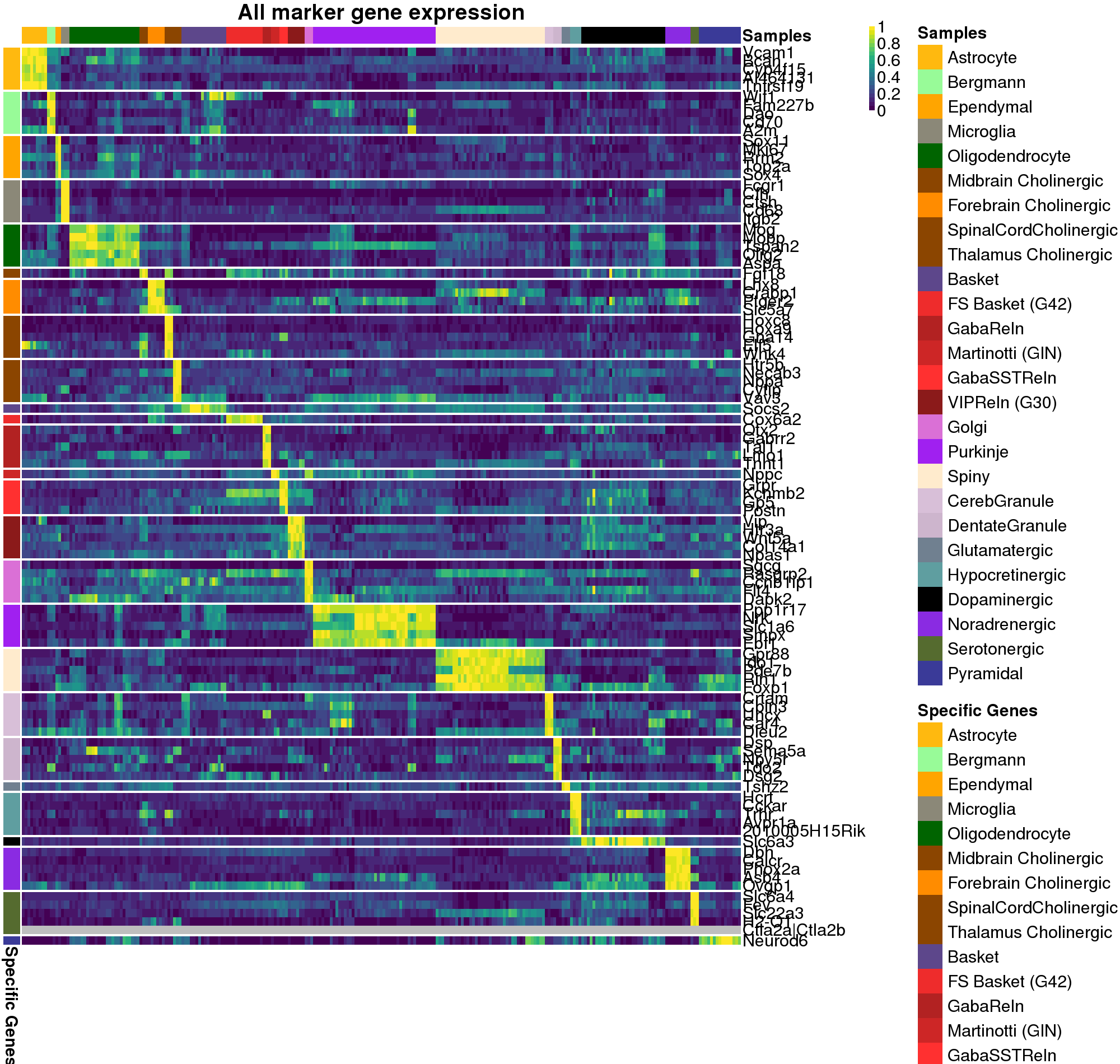

Supplement: Extended Data 3 [file enu006172455so2.zip › neuroExpressoAnalysis-master/analysis/01.SelectGenes/GenePlotsTop/All.png]

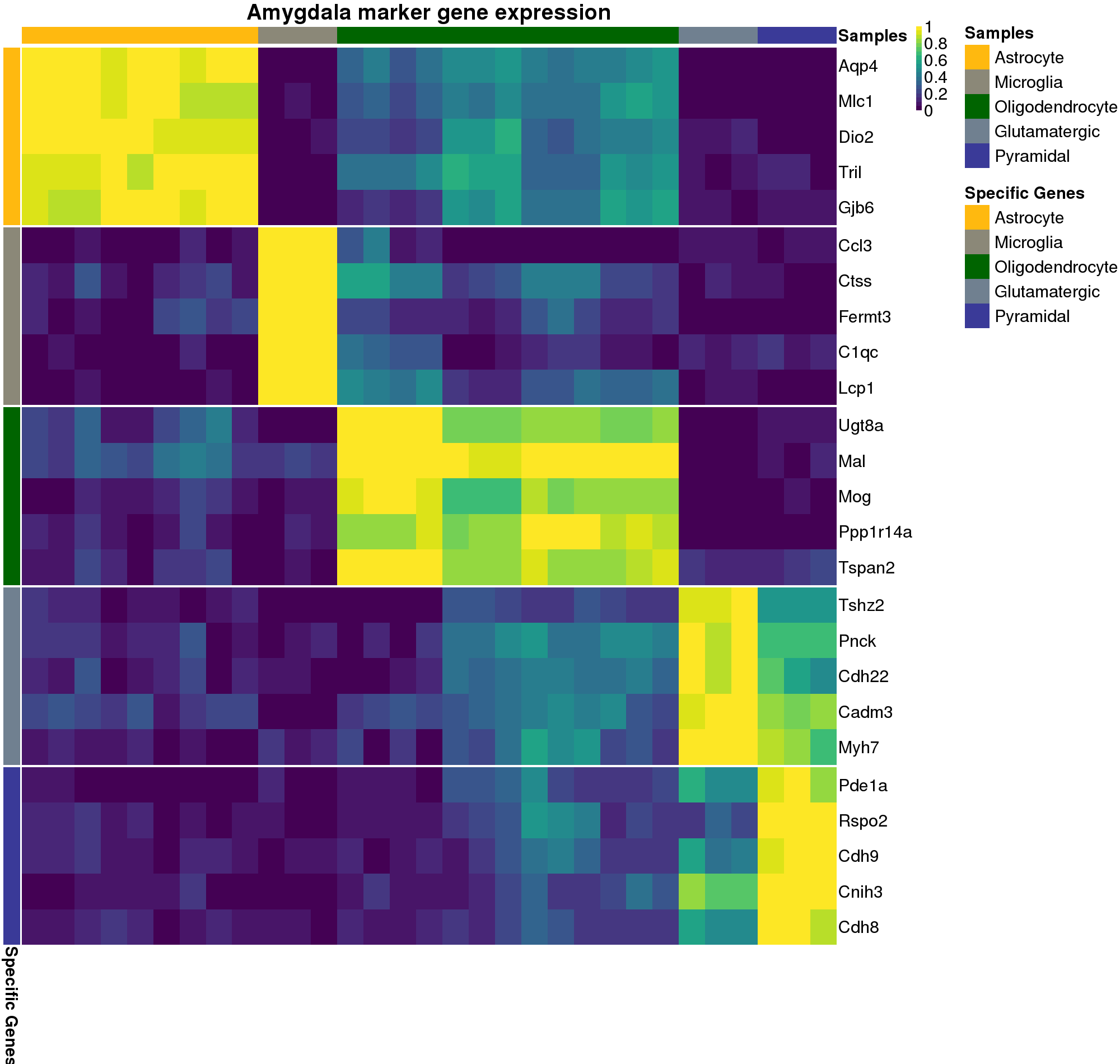

Supplement: Extended Data 3 [file enu006172455so2.zip › neuroExpressoAnalysis-master/analysis/01.SelectGenes/GenePlotsTop/Amygdala.png]

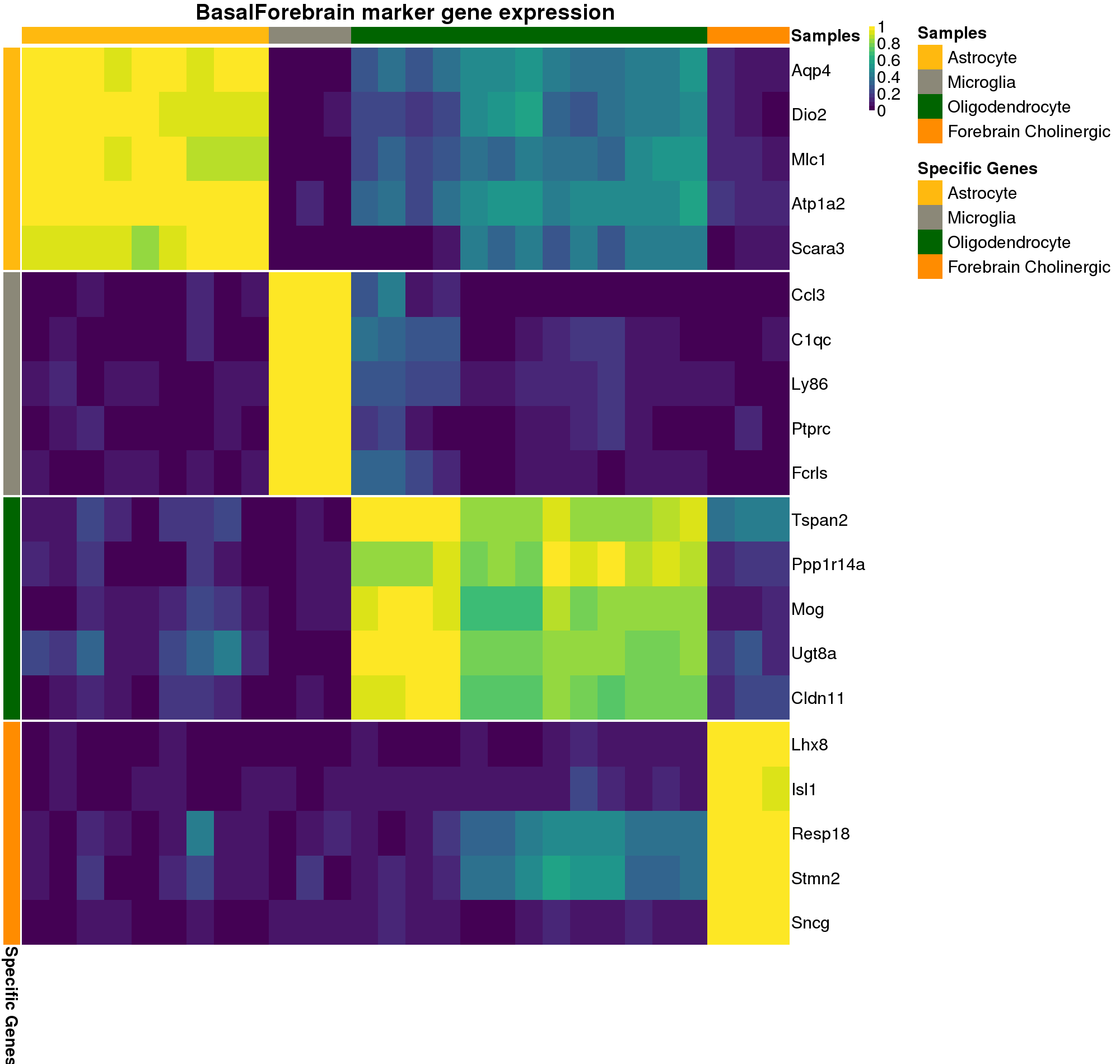

Supplement: Extended Data 3 [file enu006172455so2.zip › neuroExpressoAnalysis-master/analysis/01.SelectGenes/GenePlotsTop/BasalForebrain.png]

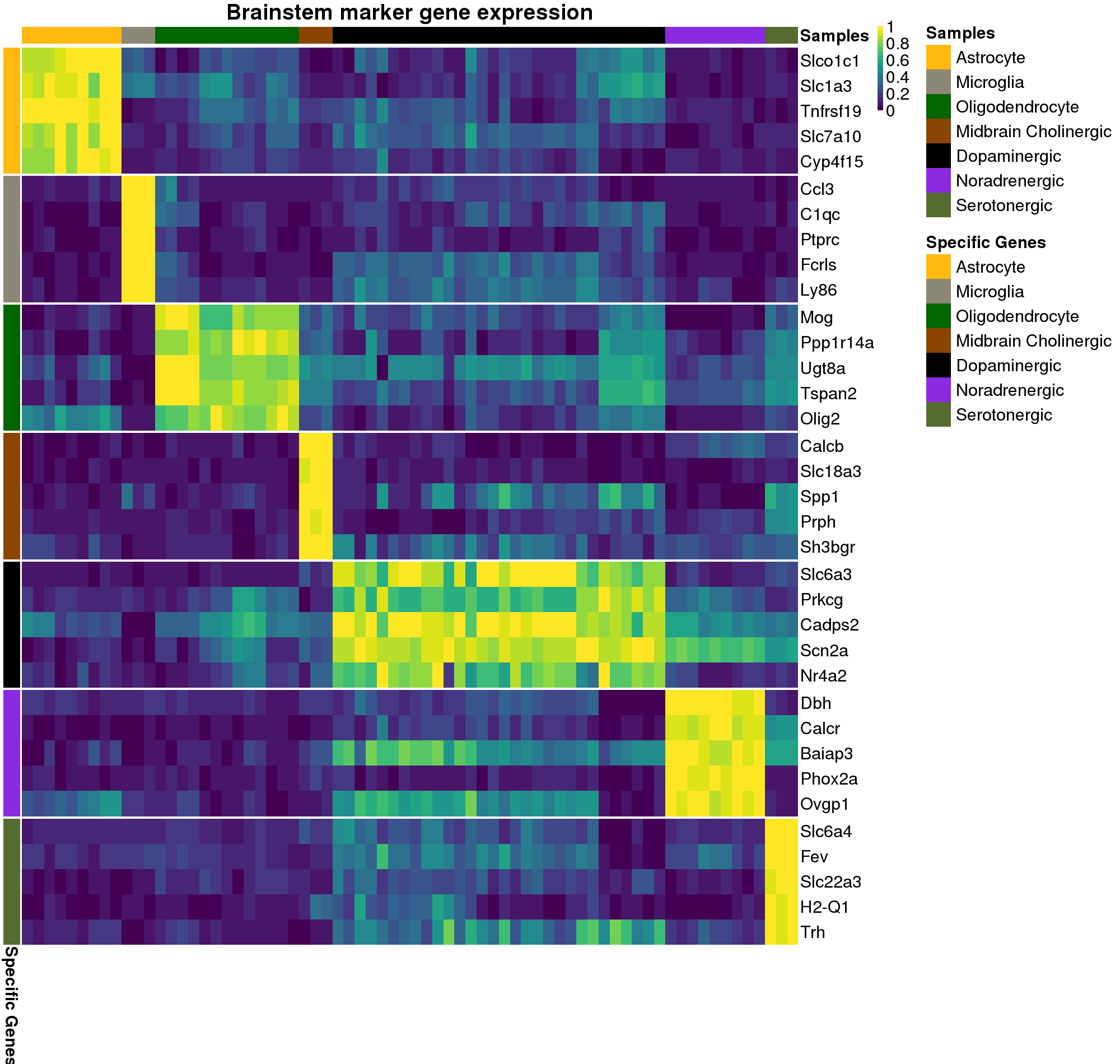

Supplement: Extended Data 3 [file enu006172455so2.zip › neuroExpressoAnalysis-master/analysis/01.SelectGenes/GenePlotsTop/Brainstem.png]

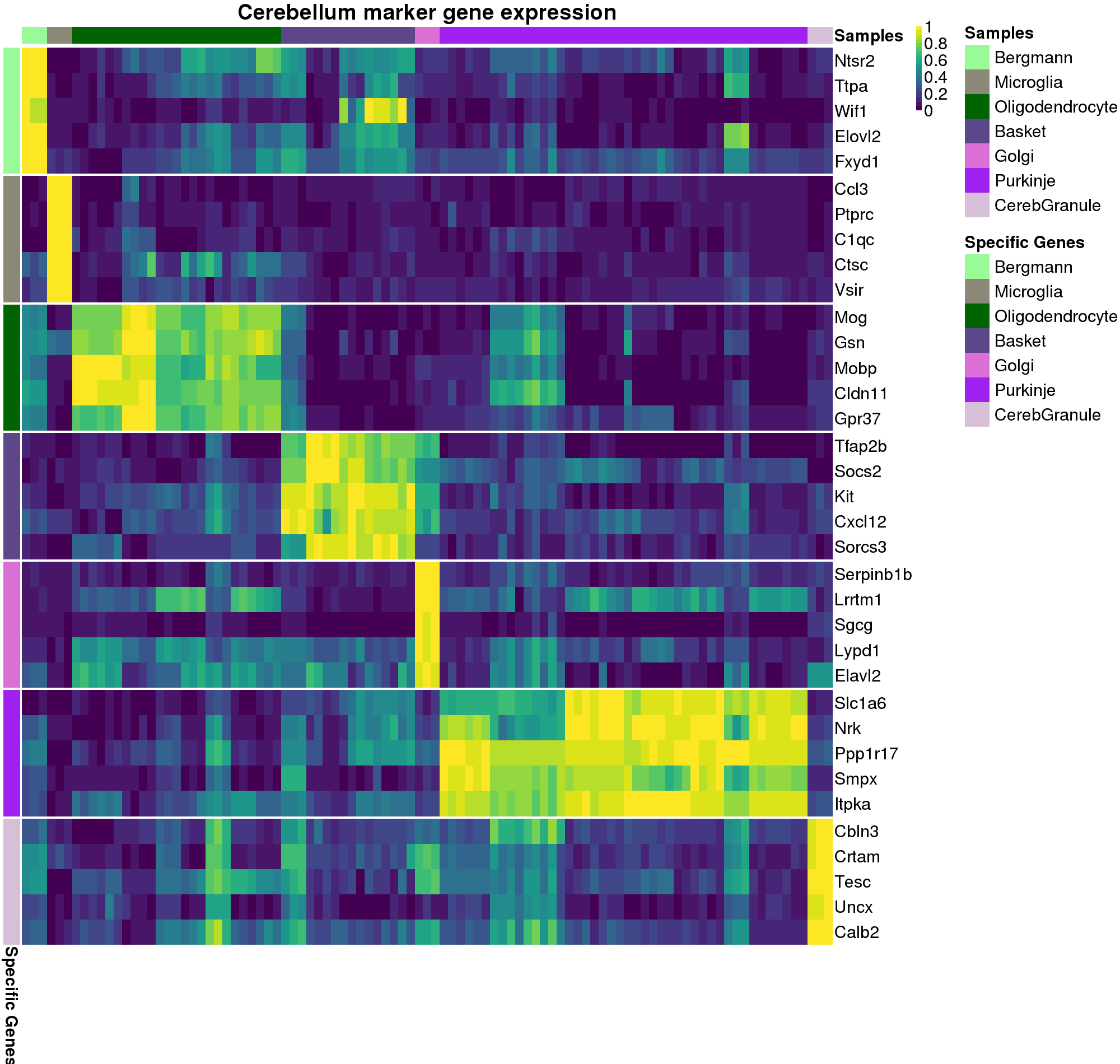

Supplement: Extended Data 3 [file enu006172455so2.zip › neuroExpressoAnalysis-master/analysis/01.SelectGenes/GenePlotsTop/Cerebellum.png]

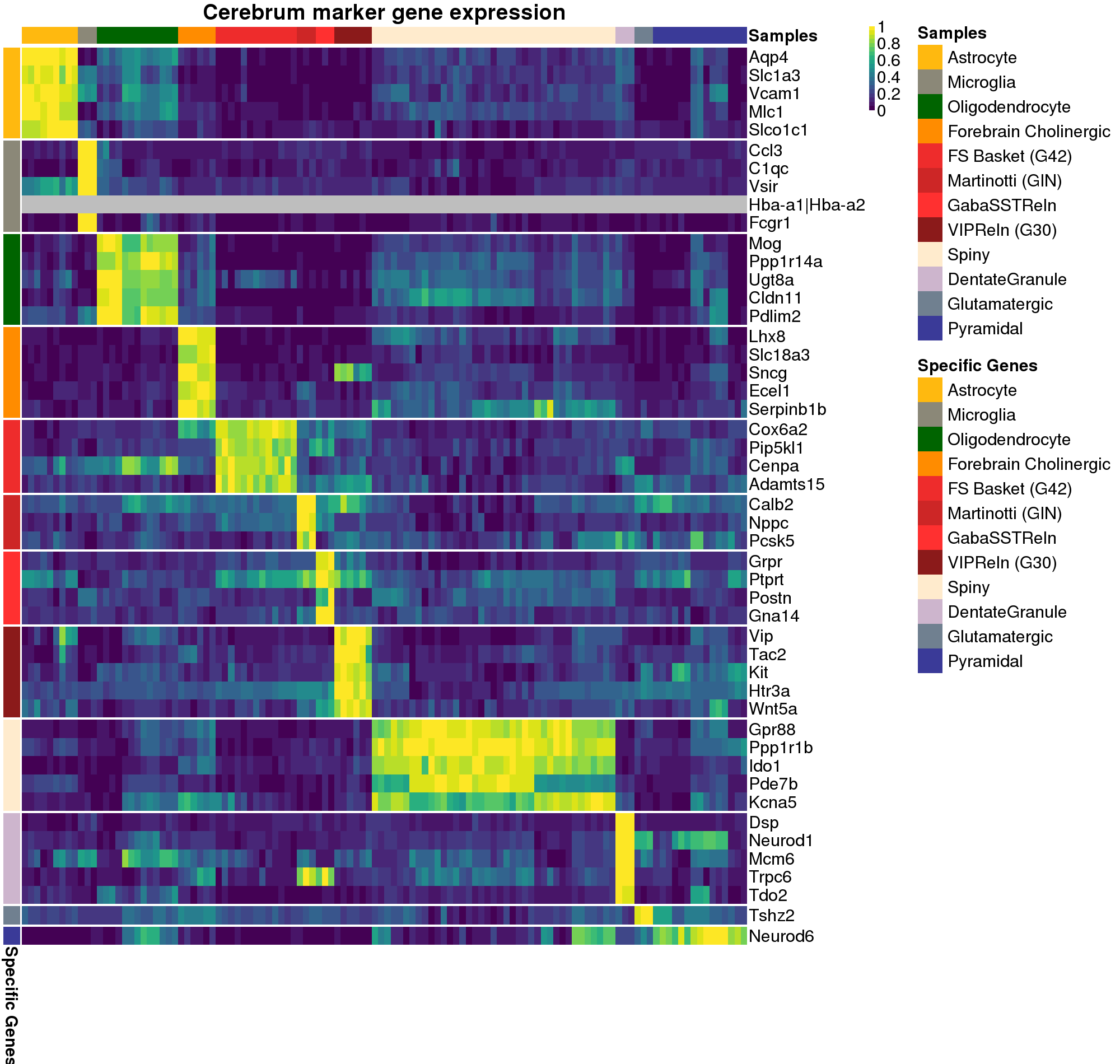

Supplement: Extended Data 3 [file enu006172455so2.zip › neuroExpressoAnalysis-master/analysis/01.SelectGenes/GenePlotsTop/Cerebrum.png]

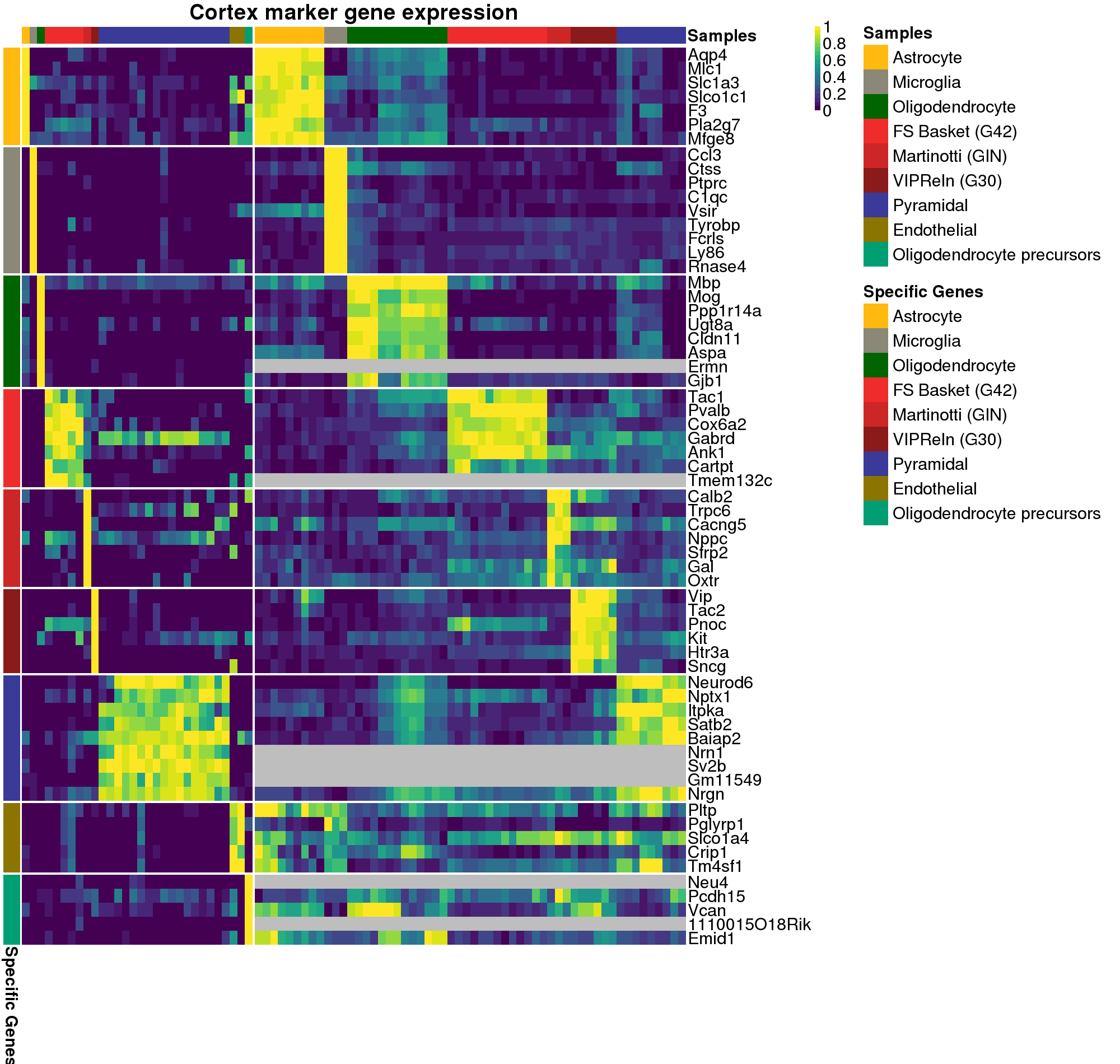

Supplement: Extended Data 3 [file enu006172455so2.zip › neuroExpressoAnalysis-master/analysis/01.SelectGenes/GenePlotsTop/Cortex.png]

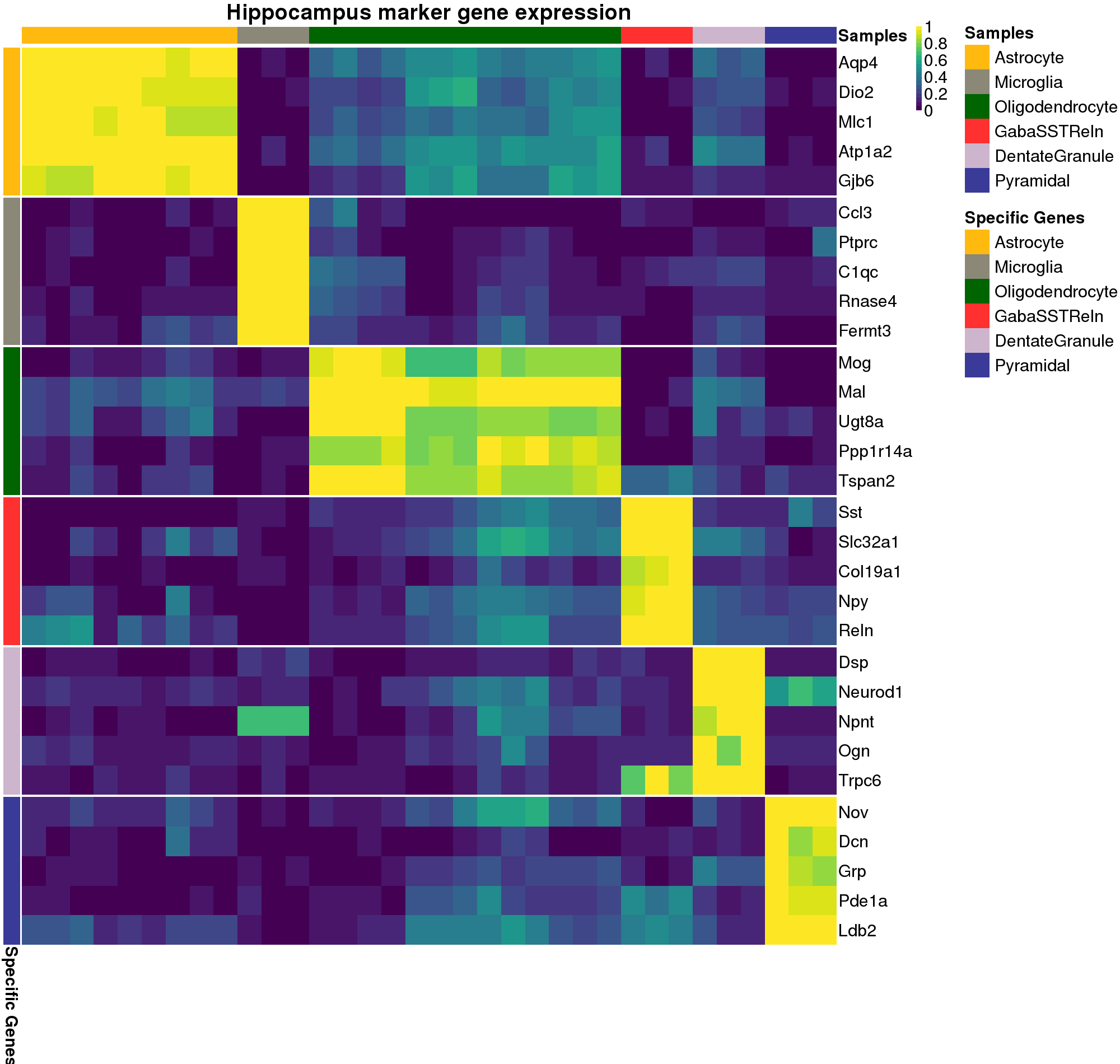

Supplement: Extended Data 3 [file enu006172455so2.zip › neuroExpressoAnalysis-master/analysis/01.SelectGenes/GenePlotsTop/Hippocampus.png]

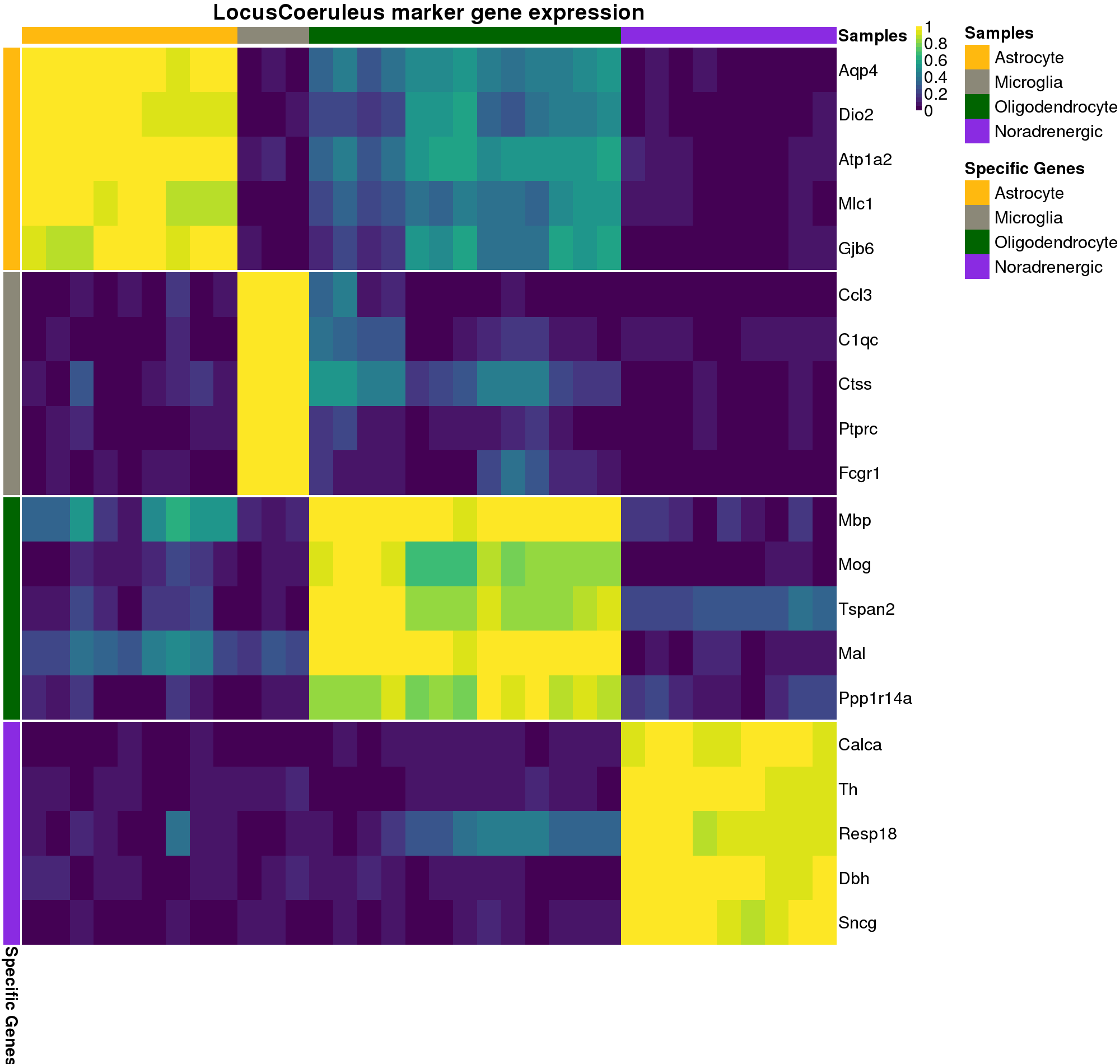

Supplement: Extended Data 3 [file enu006172455so2.zip › neuroExpressoAnalysis-master/analysis/01.SelectGenes/GenePlotsTop/LocusCoeruleus.png]

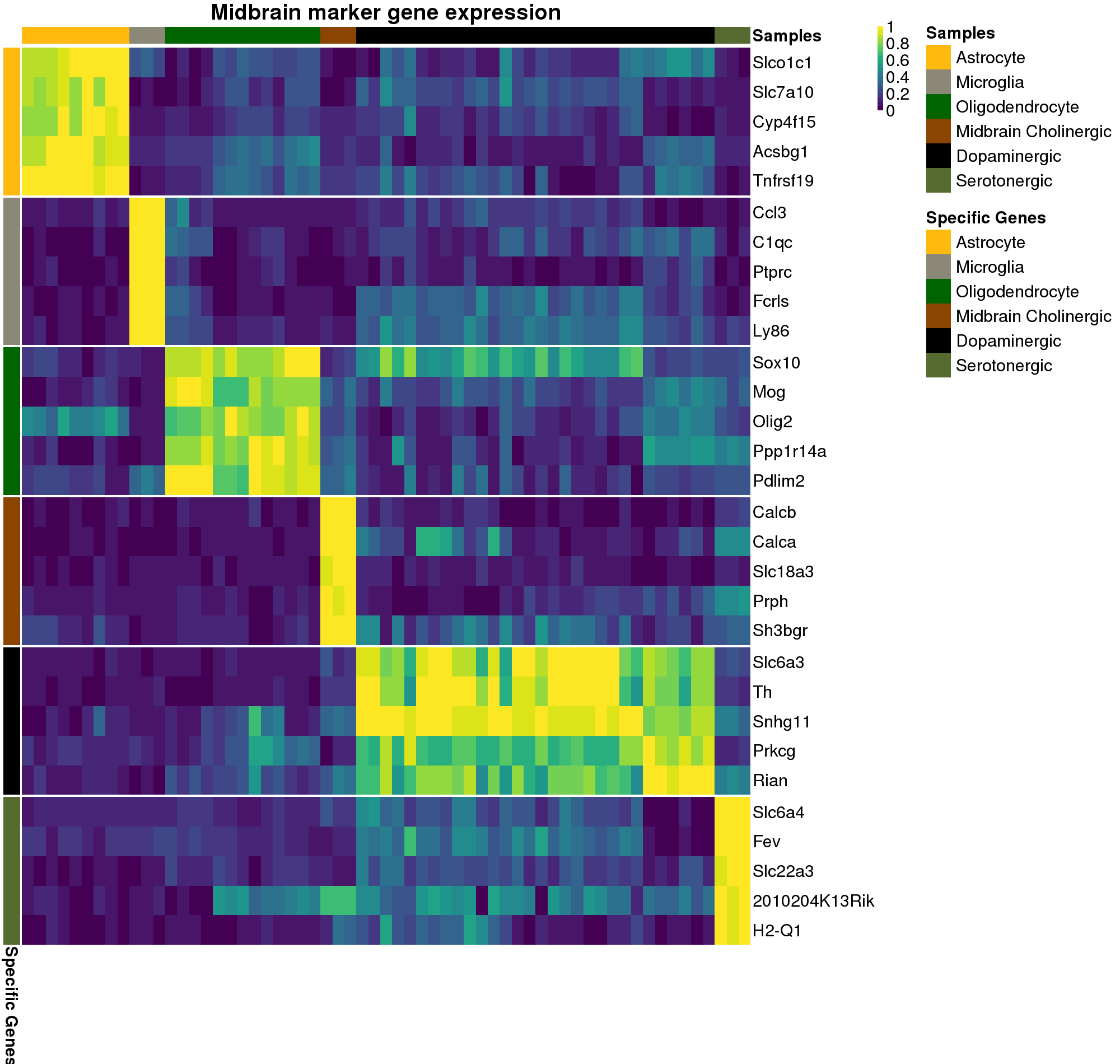

Supplement: Extended Data 3 [file enu006172455so2.zip › neuroExpressoAnalysis-master/analysis/01.SelectGenes/GenePlotsTop/Midbrain.png]

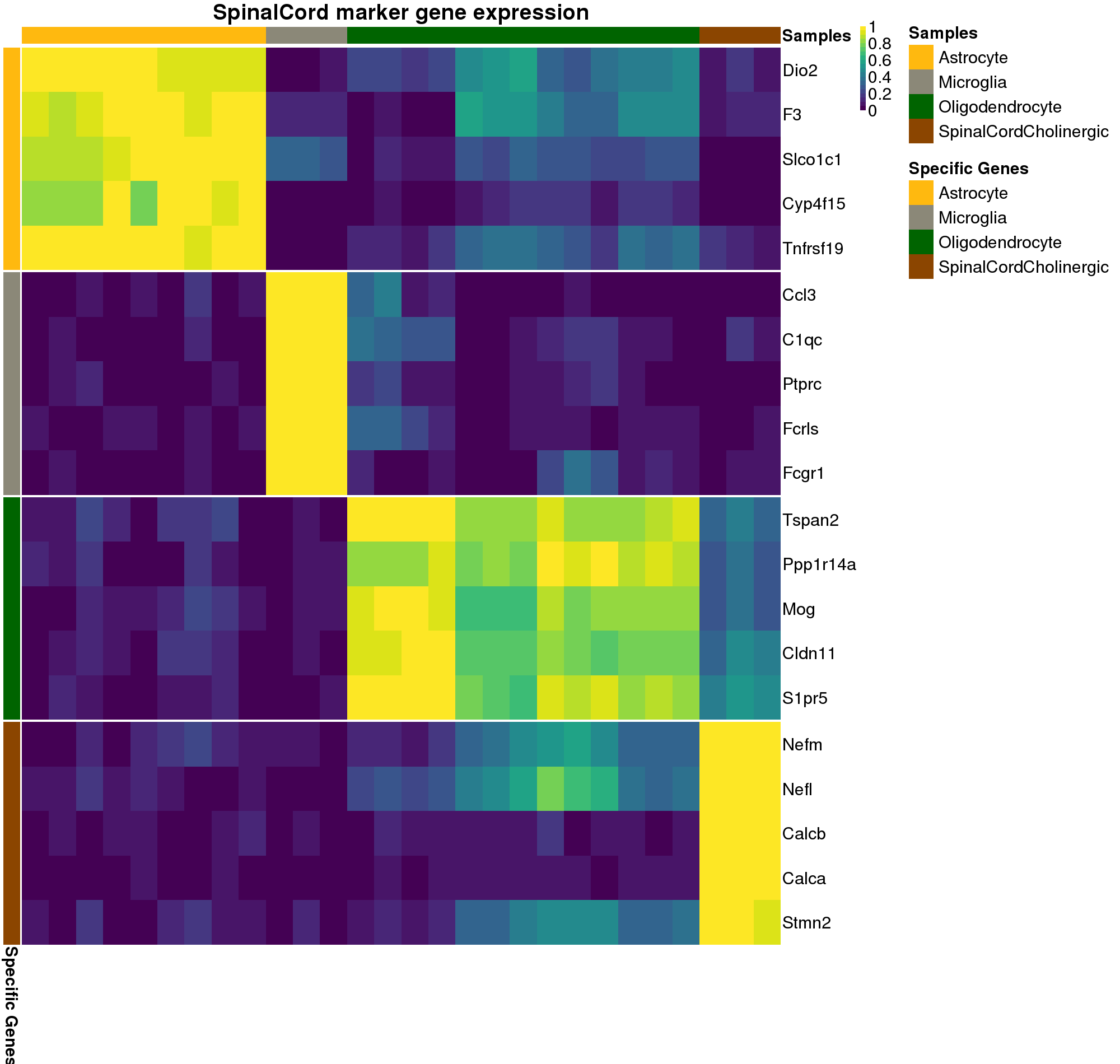

Supplement: Extended Data 3 [file enu006172455so2.zip › neuroExpressoAnalysis-master/analysis/01.SelectGenes/GenePlotsTop/SpinalCord.png]

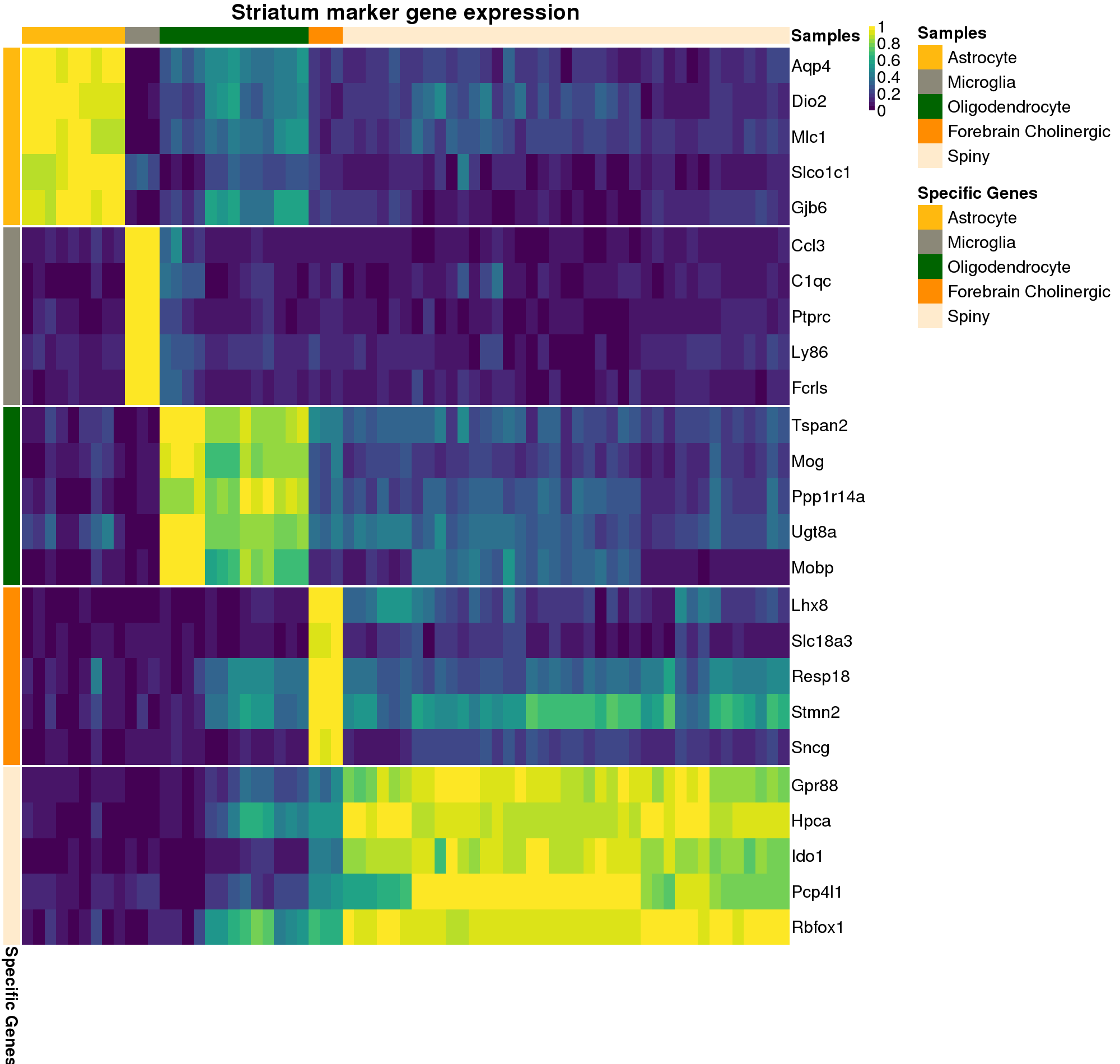

Supplement: Extended Data 3 [file enu006172455so2.zip › neuroExpressoAnalysis-master/analysis/01.SelectGenes/GenePlotsTop/Striatum.png]

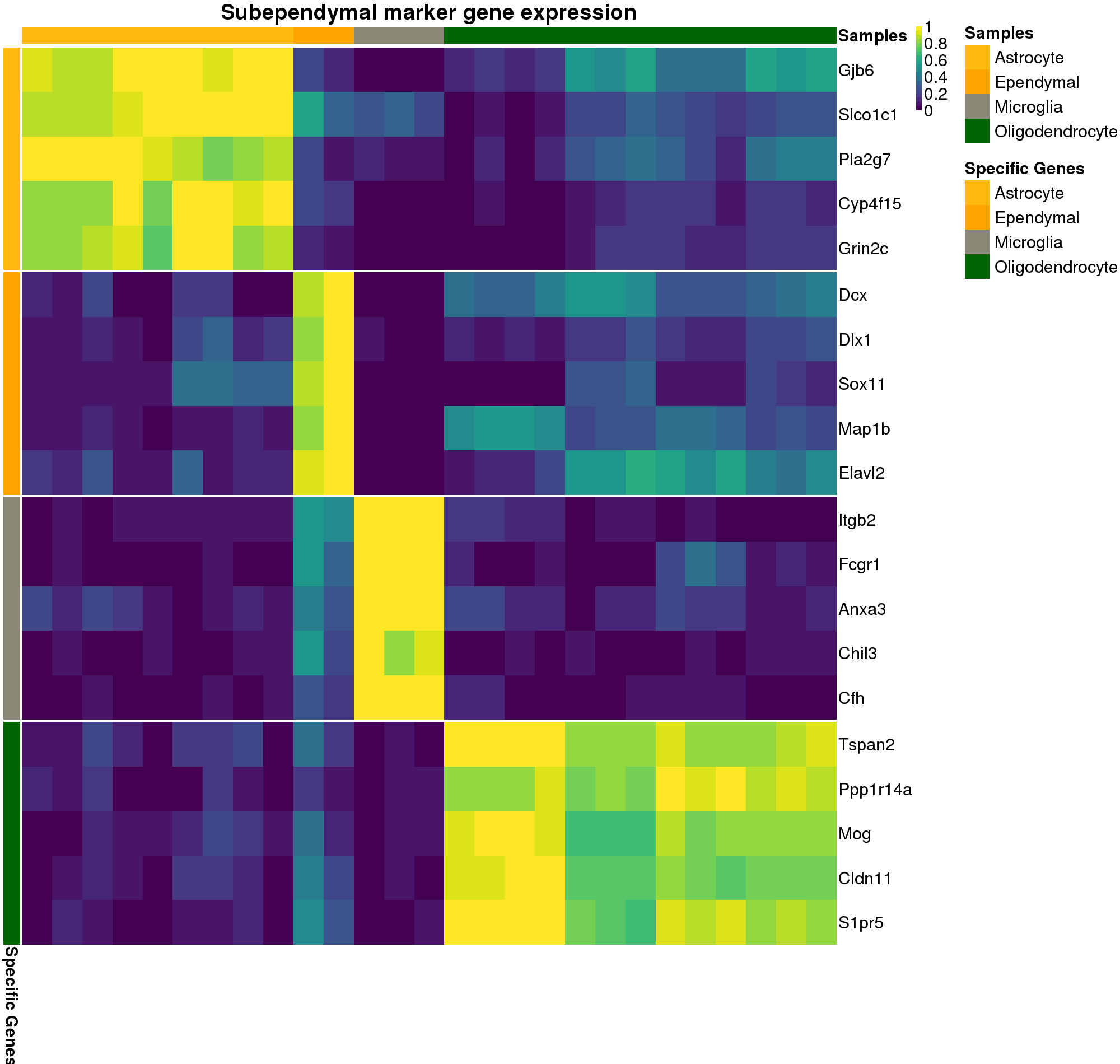

Supplement: Extended Data 3 [file enu006172455so2.zip › neuroExpressoAnalysis-master/analysis/01.SelectGenes/GenePlotsTop/Subependymal.png]

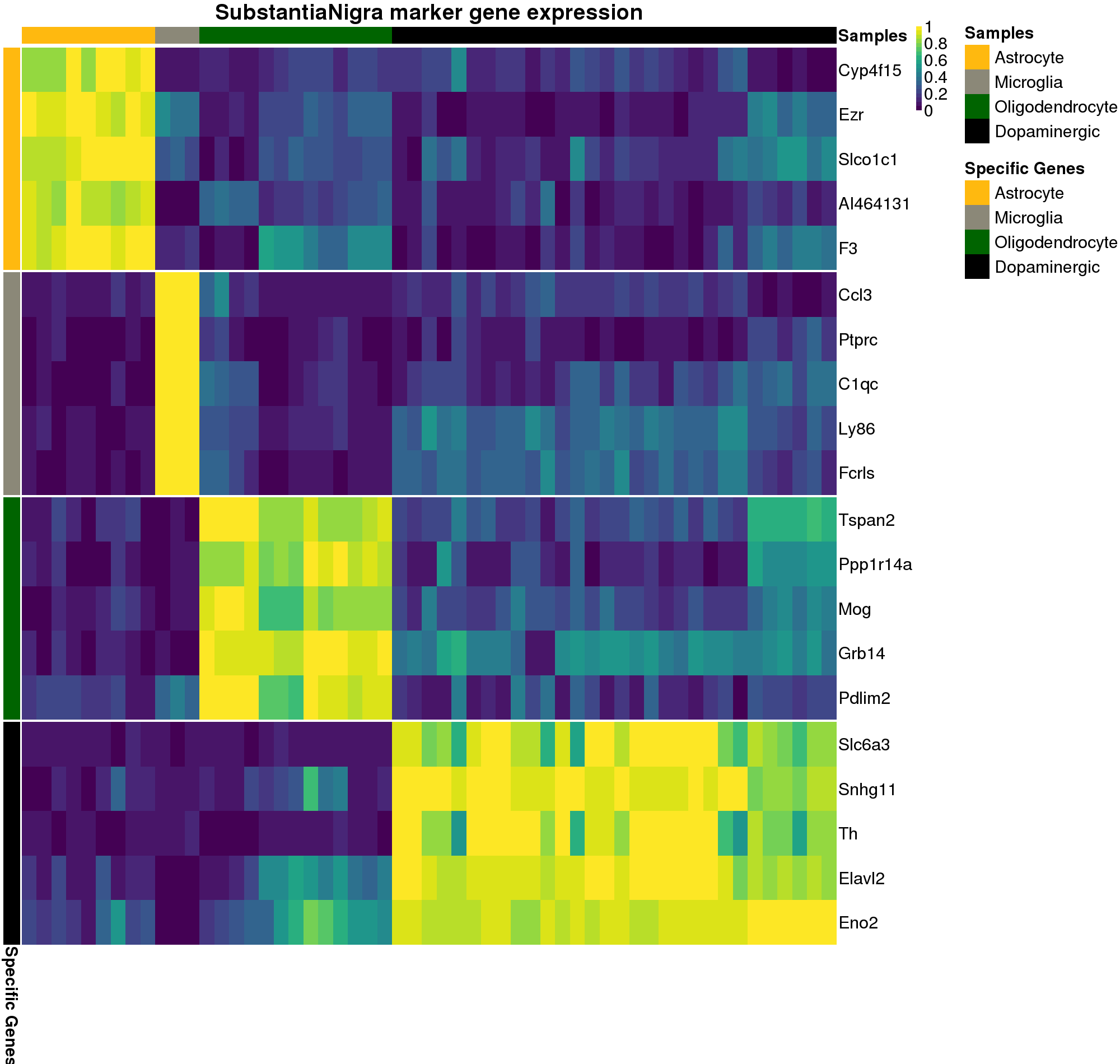

Supplement: Extended Data 3 [file enu006172455so2.zip › neuroExpressoAnalysis-master/analysis/01.SelectGenes/GenePlotsTop/SubstantiaNigra.png]

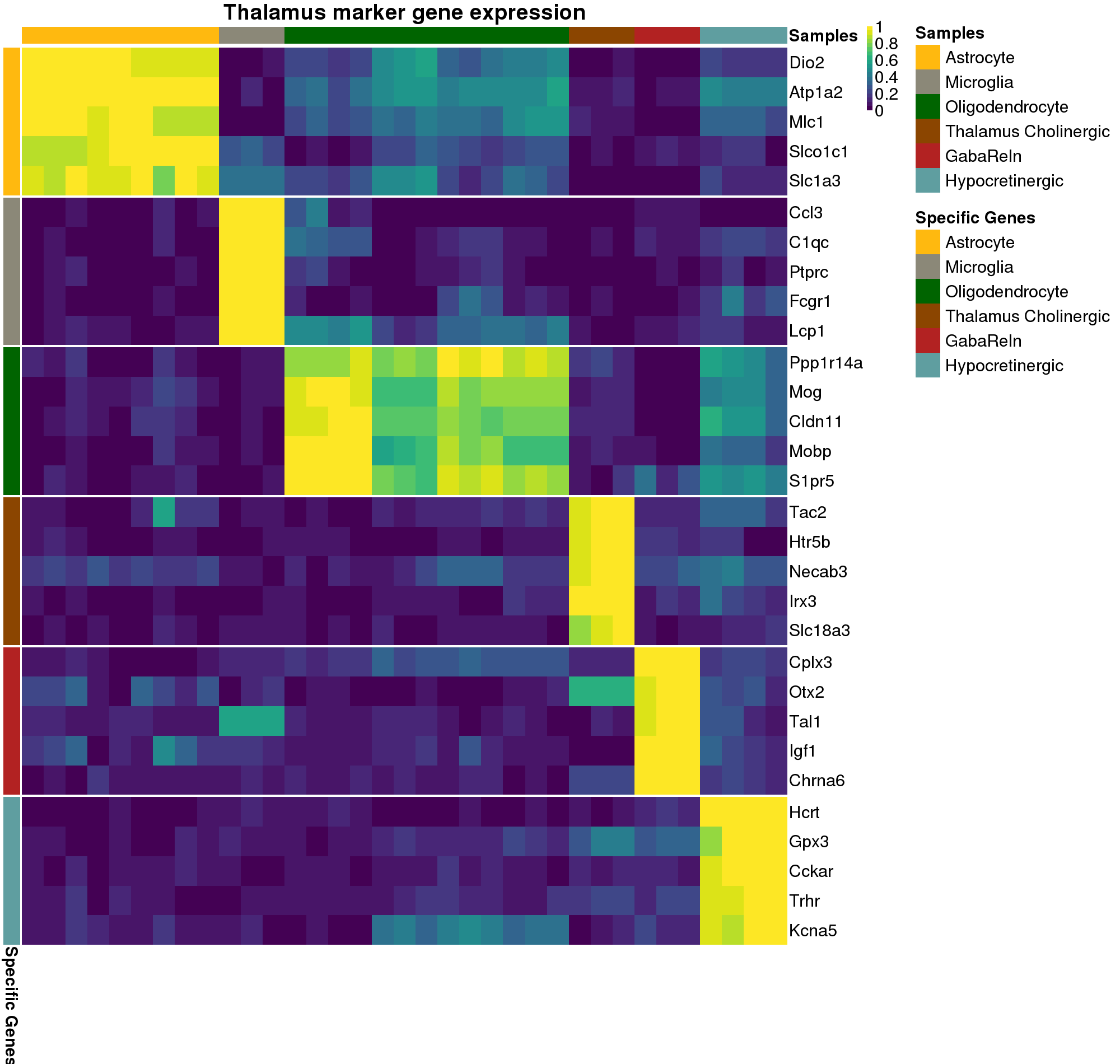

Supplement: Extended Data 3 [file enu006172455so2.zip › neuroExpressoAnalysis-master/analysis/01.SelectGenes/GenePlotsTop/Thalamus.png]
