# Supplementary material for: Cross-Laboratory Analysis of Brain Cell Type Transcriptomes with Applications to Interpretation of Bulk Tissue Data
Source: eNeuro. 2017 Nov 30;4(6):ENEURO.0212-17.2017. doi: 10.1523/ENEURO.0212-17.2017 (PMC5707795; doi:10.1523/ENEURO.0212-17.2017)
Supplement: Figure 4-1,2,3,4 [file enu006172455so1.pdf]

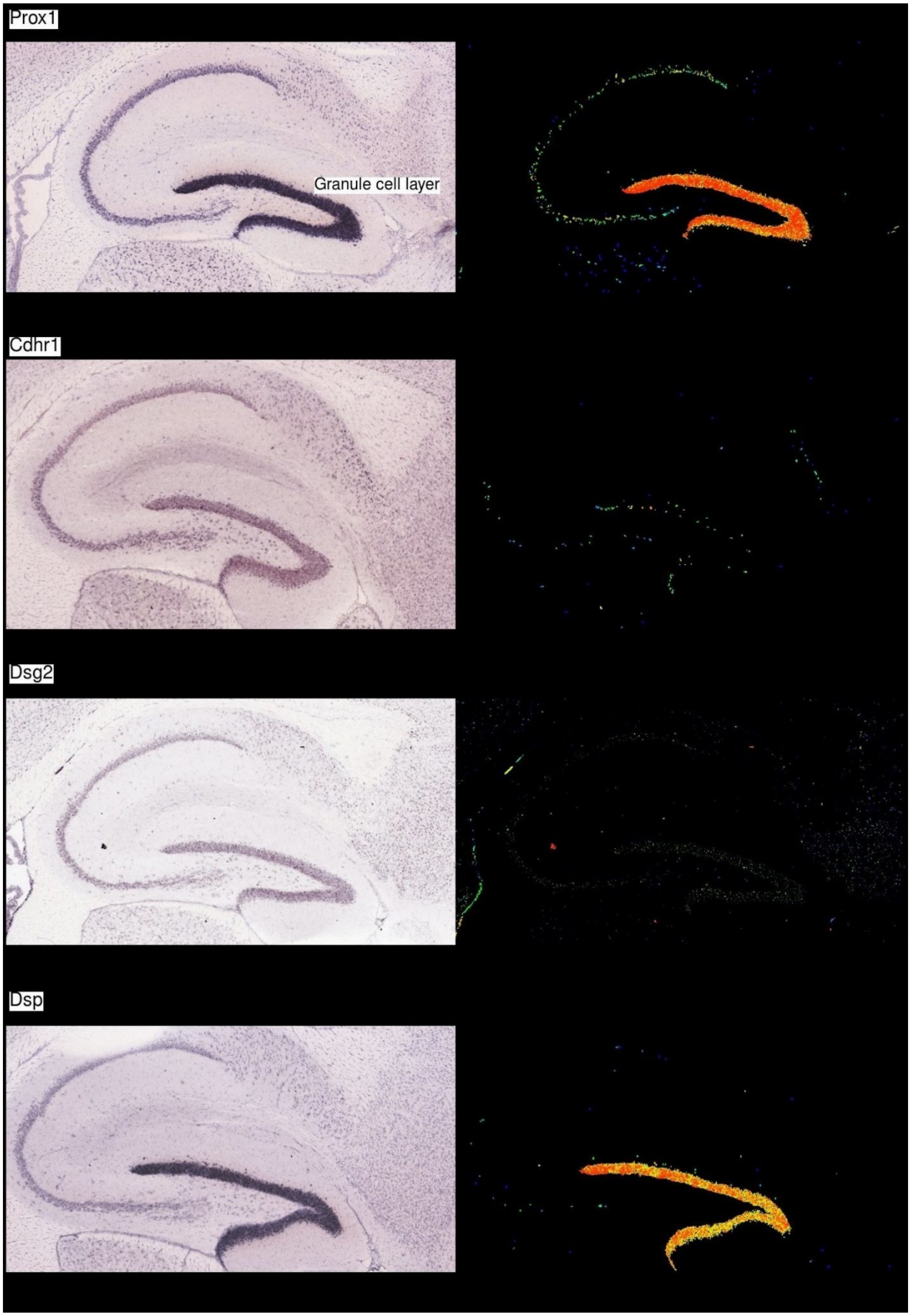


Figure 4-1: Expression of dentate granule cell markers discovered in the study in Allen Brain Atlas mouse brain in situ hybridization database. The first gene is Prox1, a known marker of dentate granule cells. The intensity is color-coded to range from blue (low expression intensity), through green (medium intensity) to red (high intensity). All images except Ogn is taken from the sagittal view. Ogn is taken from the coronal view.

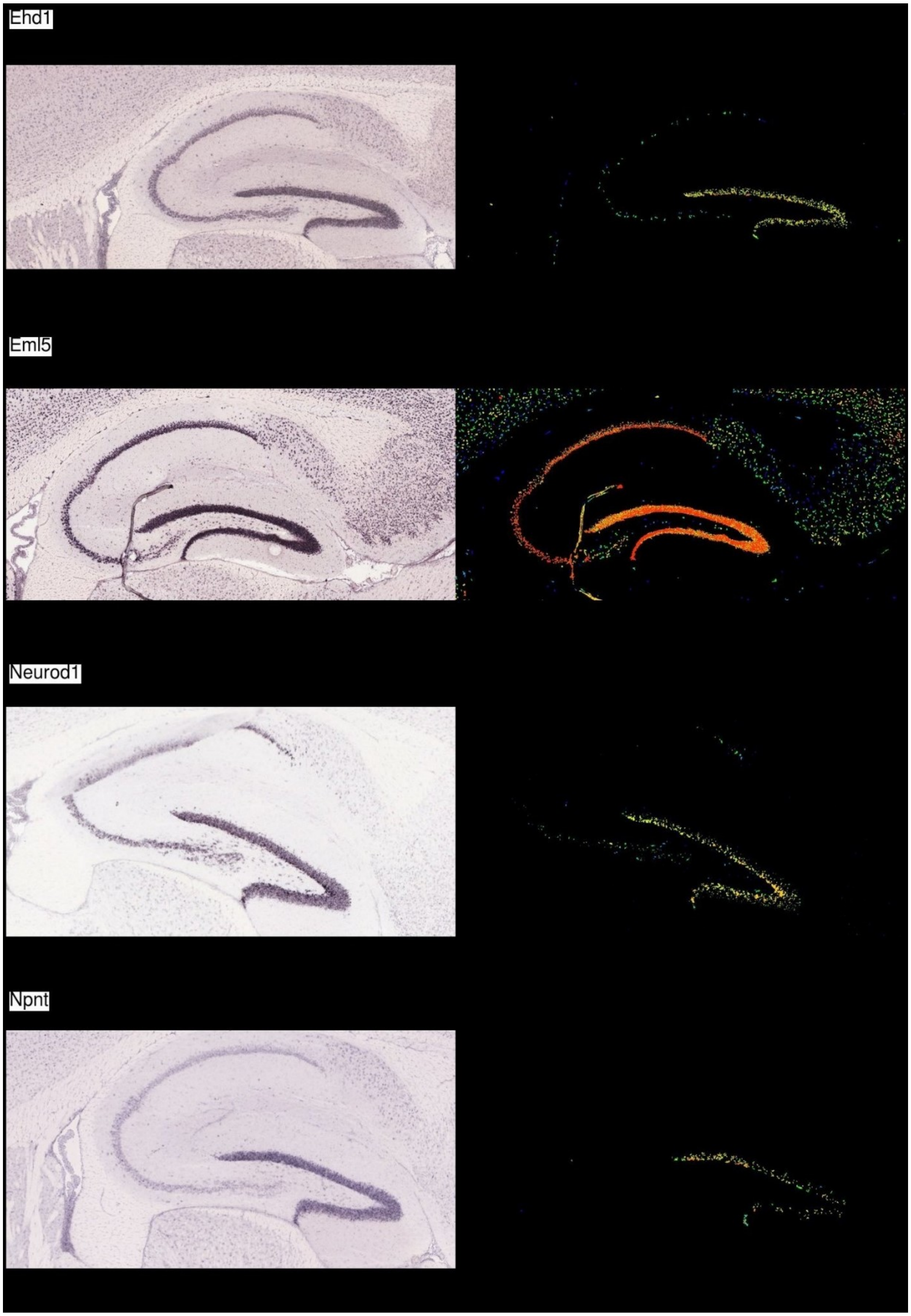


Figure 4-1 continued.

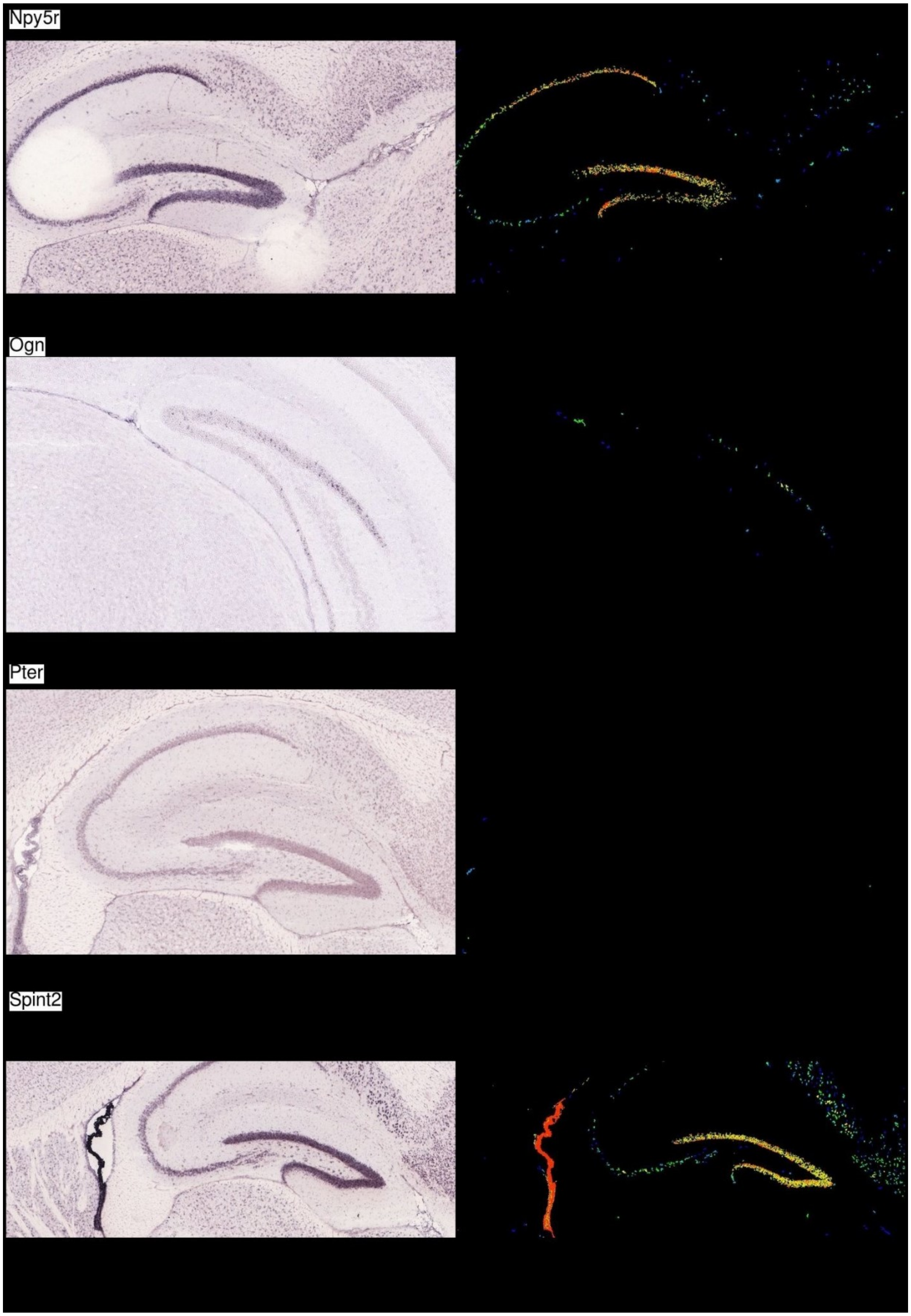


Figure 4-1 continued.

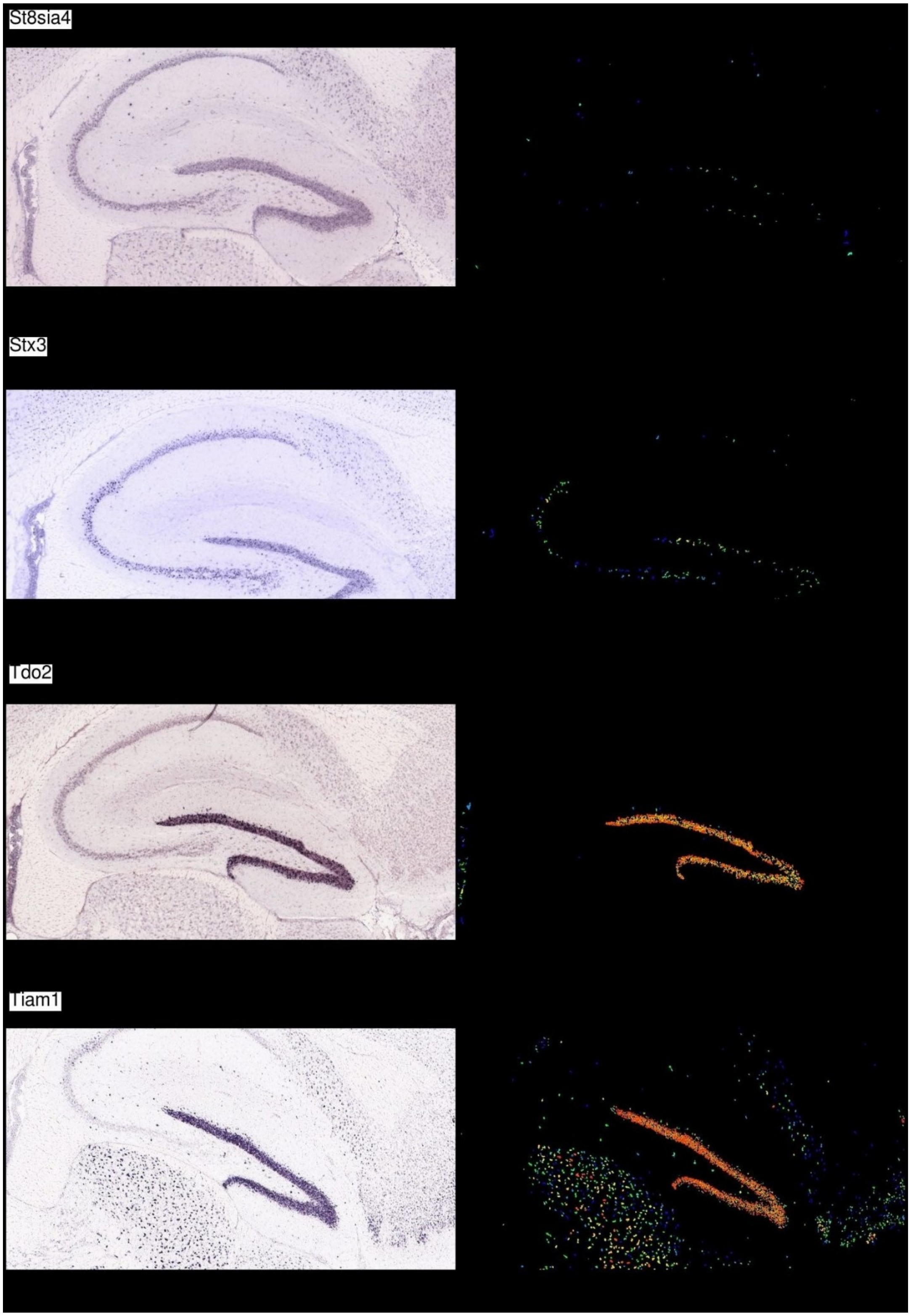


Figure 4-1 continued.

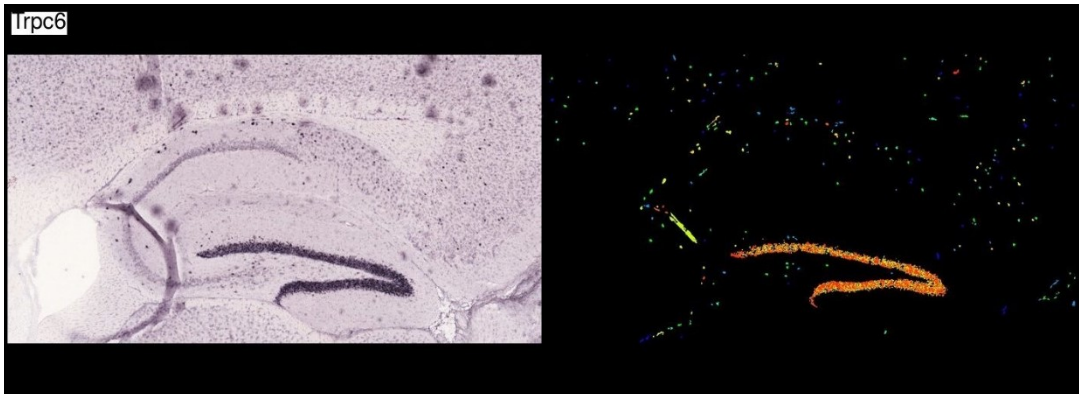


Figure 4-1 continued.

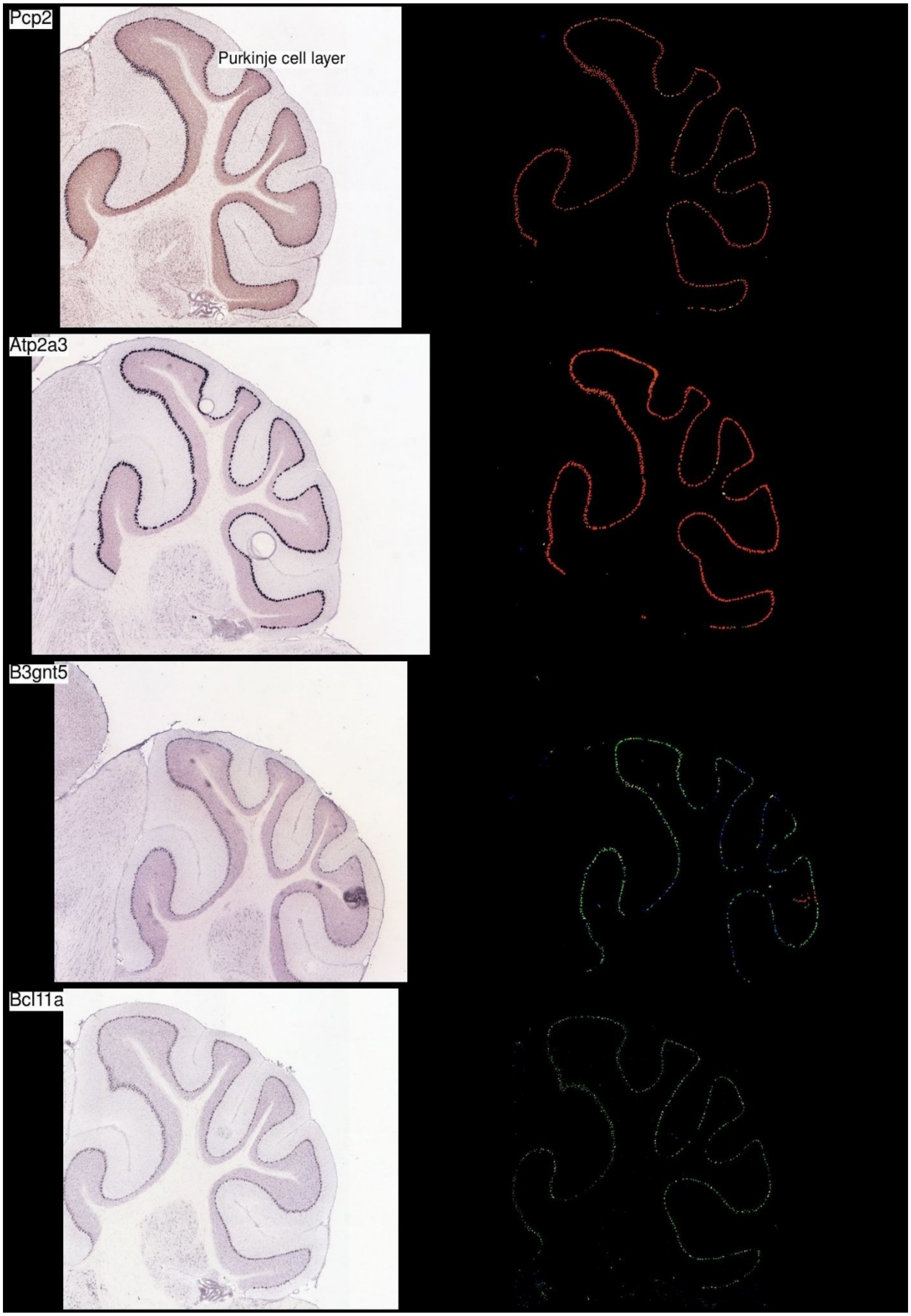


Figure 4-2: Expression of Purkinje markers discovered in the study in Allen Brain Atlas mouse brain in situ hybridization database. The first gene is Pcp2, a known marker of Purkinje cells. The intensity is color-coded to range from blue (low expression intensity), through green (medium intensity) to red (high intensity).} All images are taken from the sagittal view.

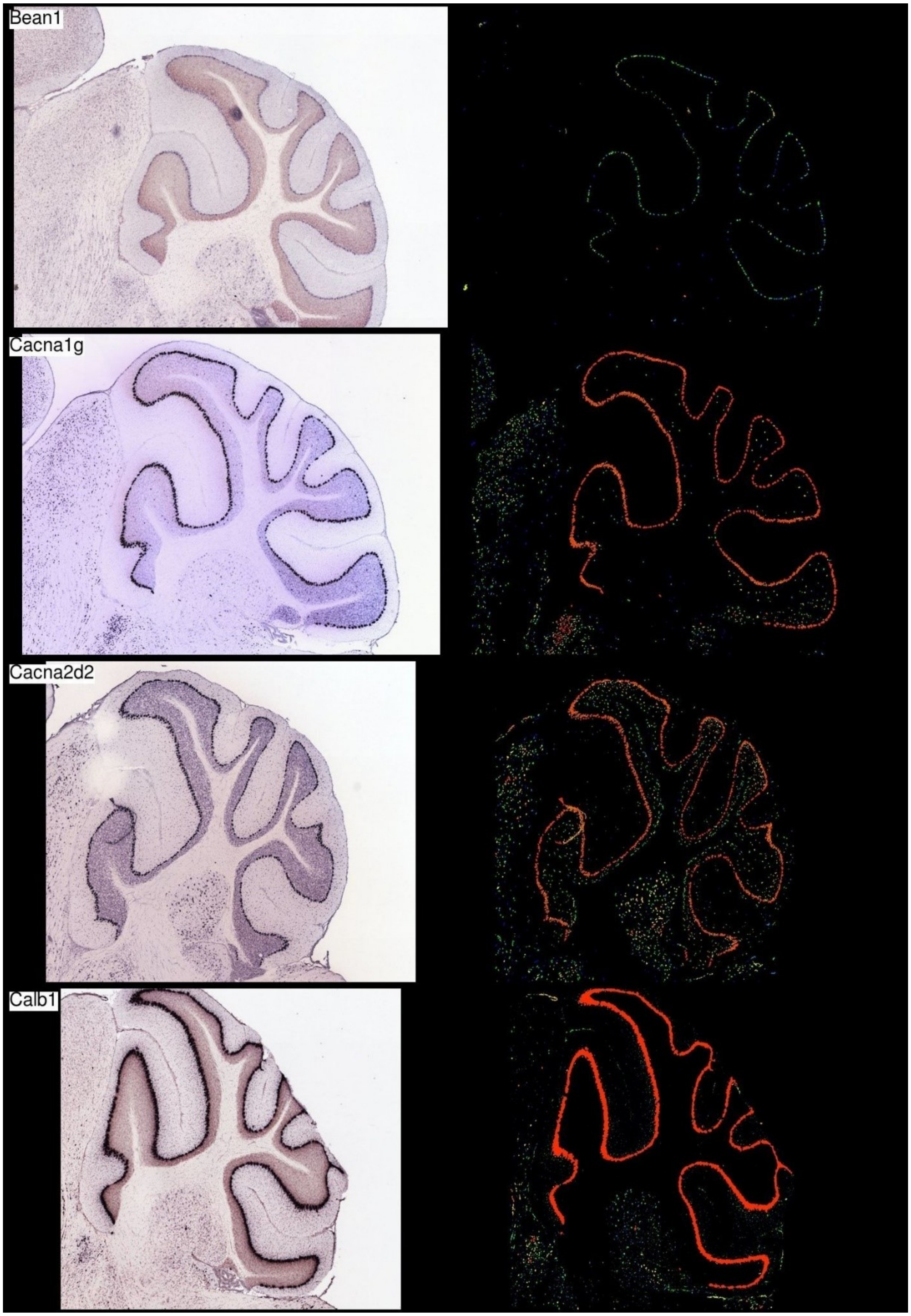


Figure 4-2 continued.

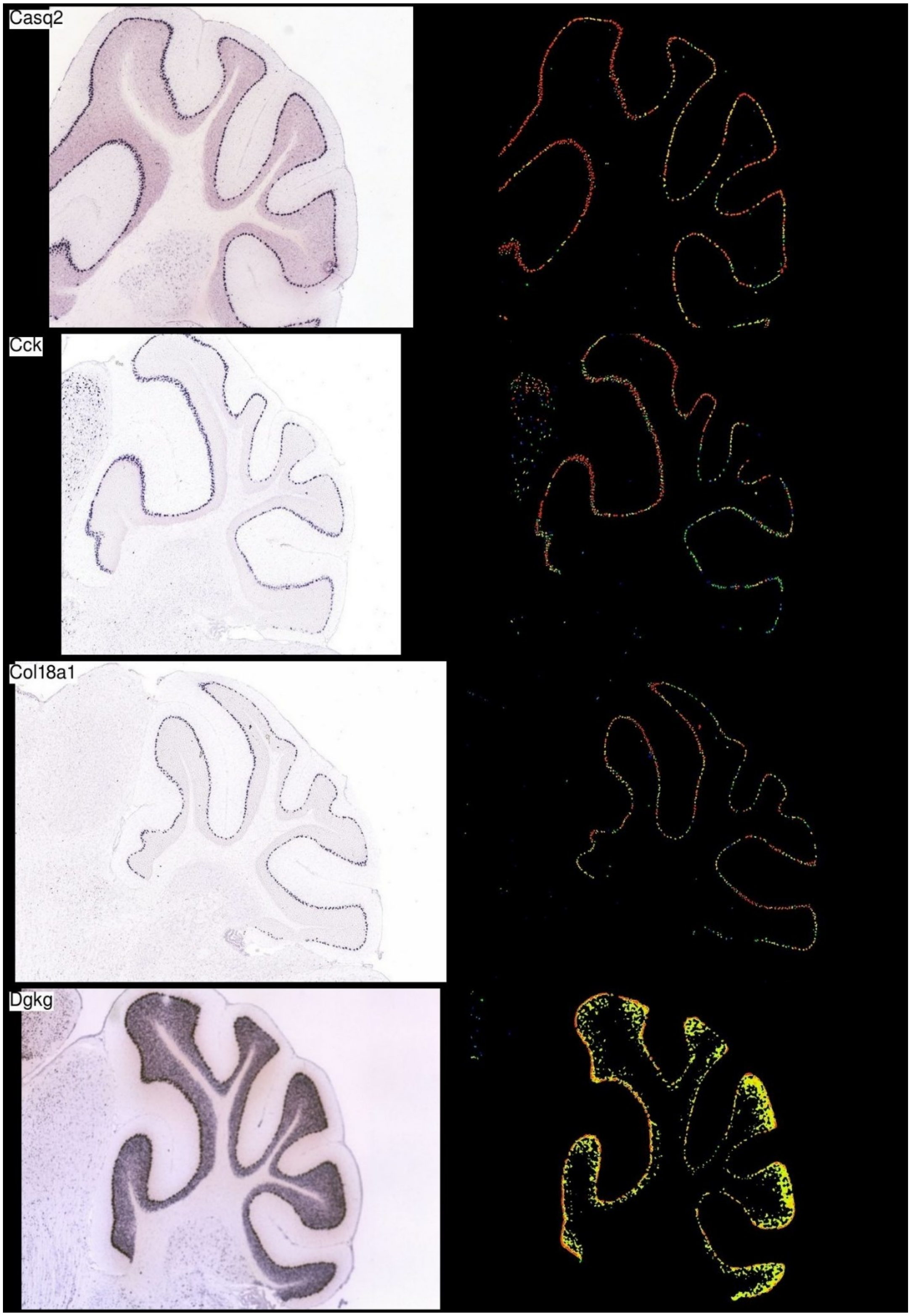


Figure 4-2 continued.

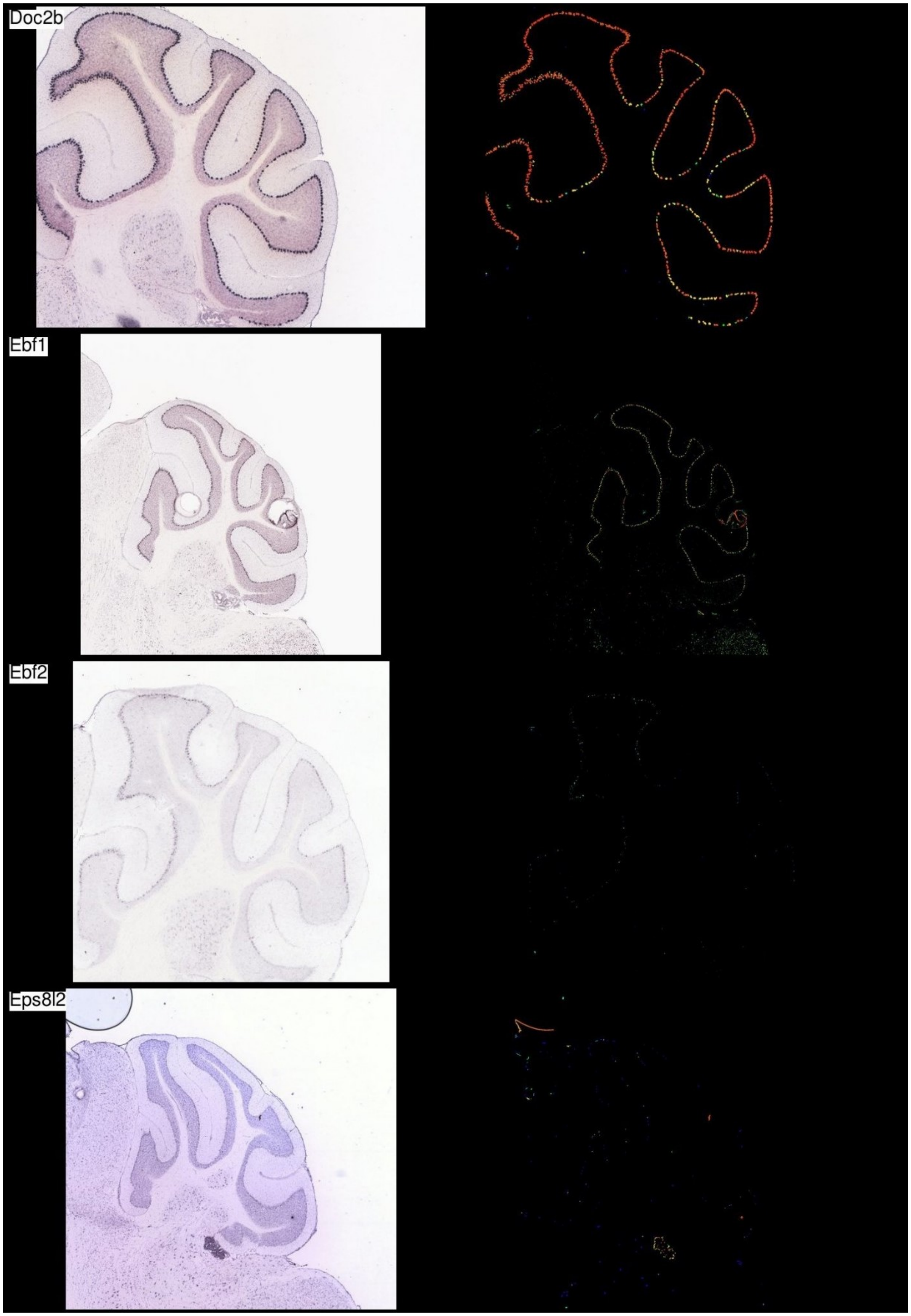


Figure 4-2 continued.

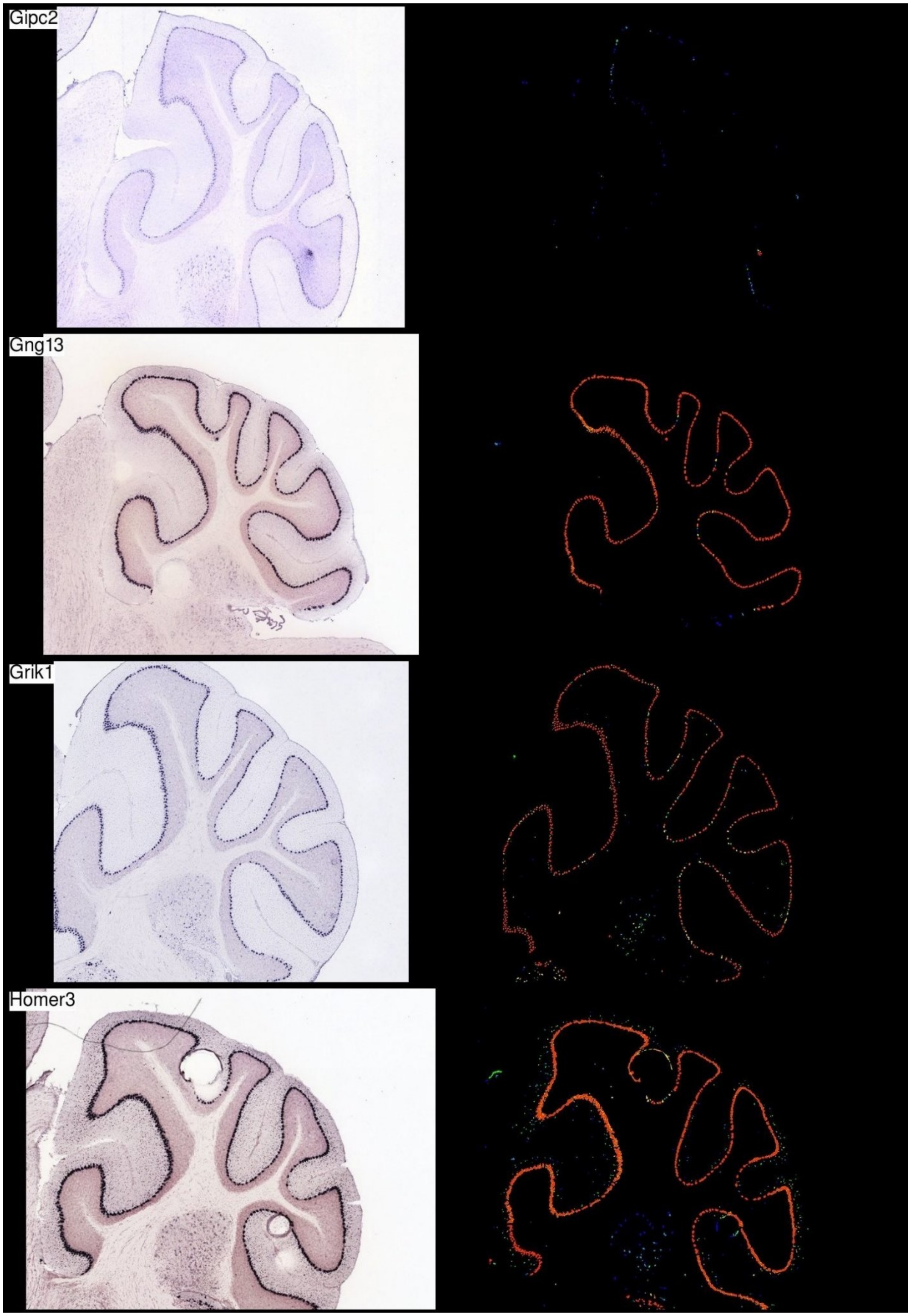


Figure 4-2 continued.

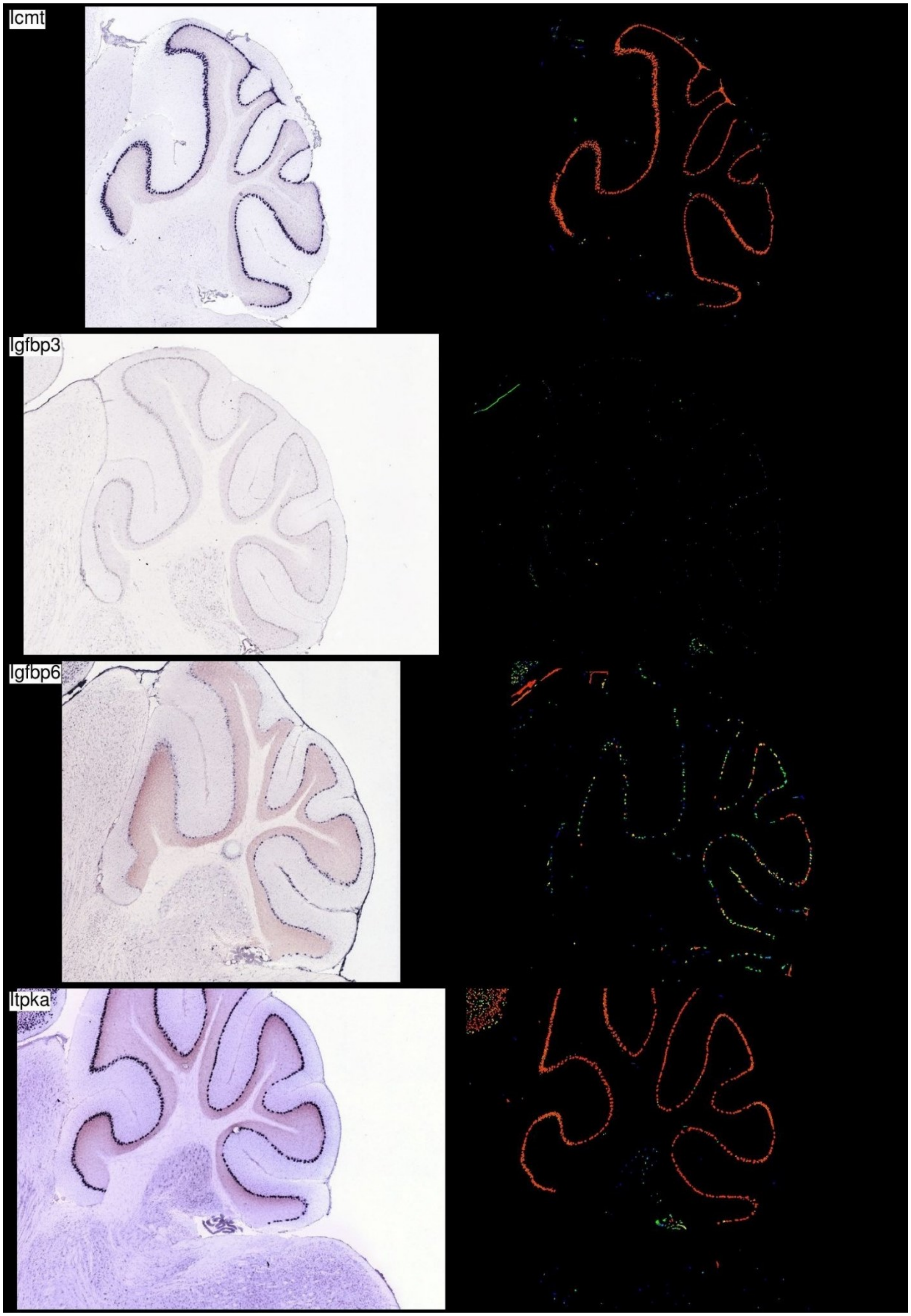


Figure 4-2 continued.

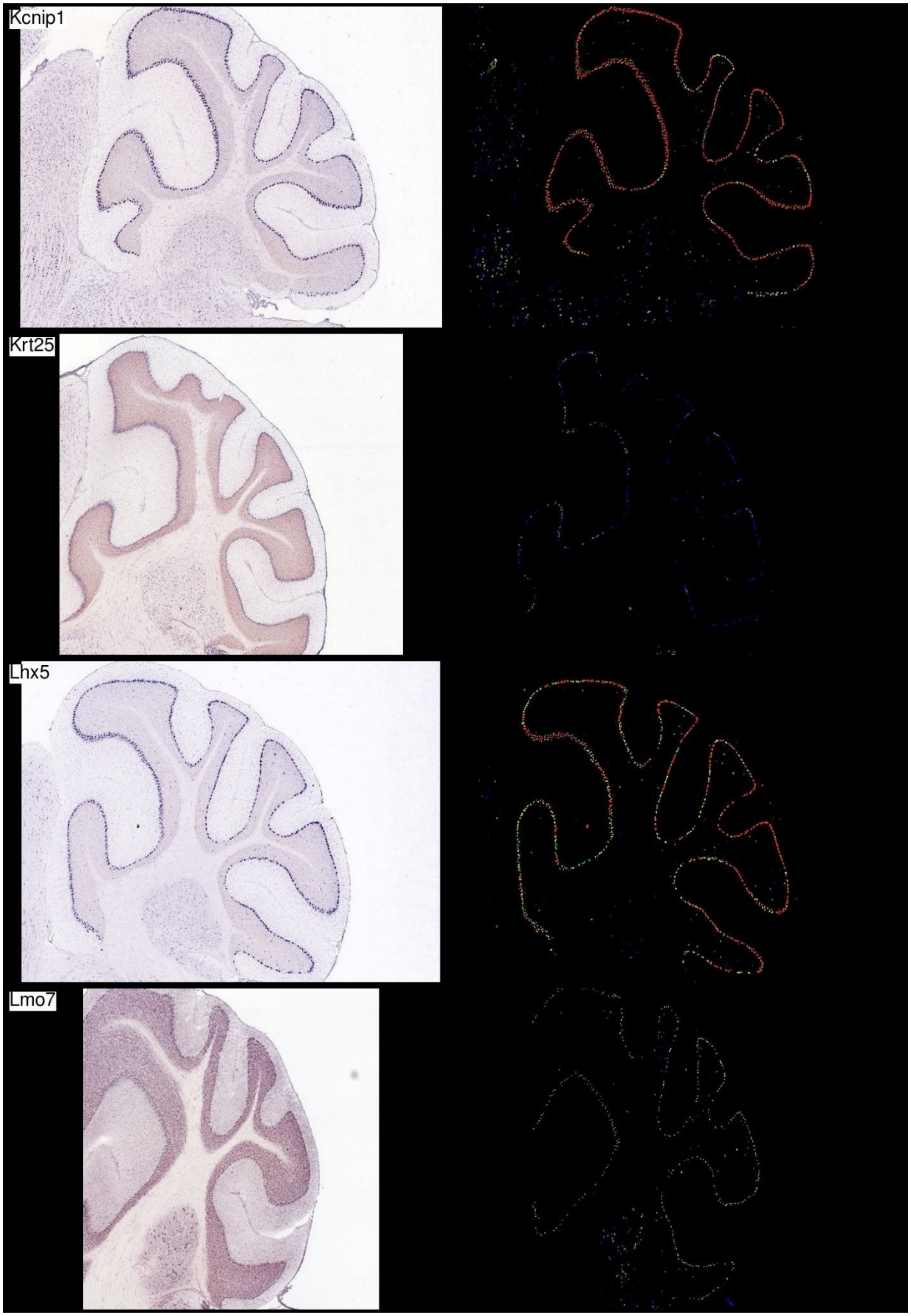


Figure 4-2 continued.

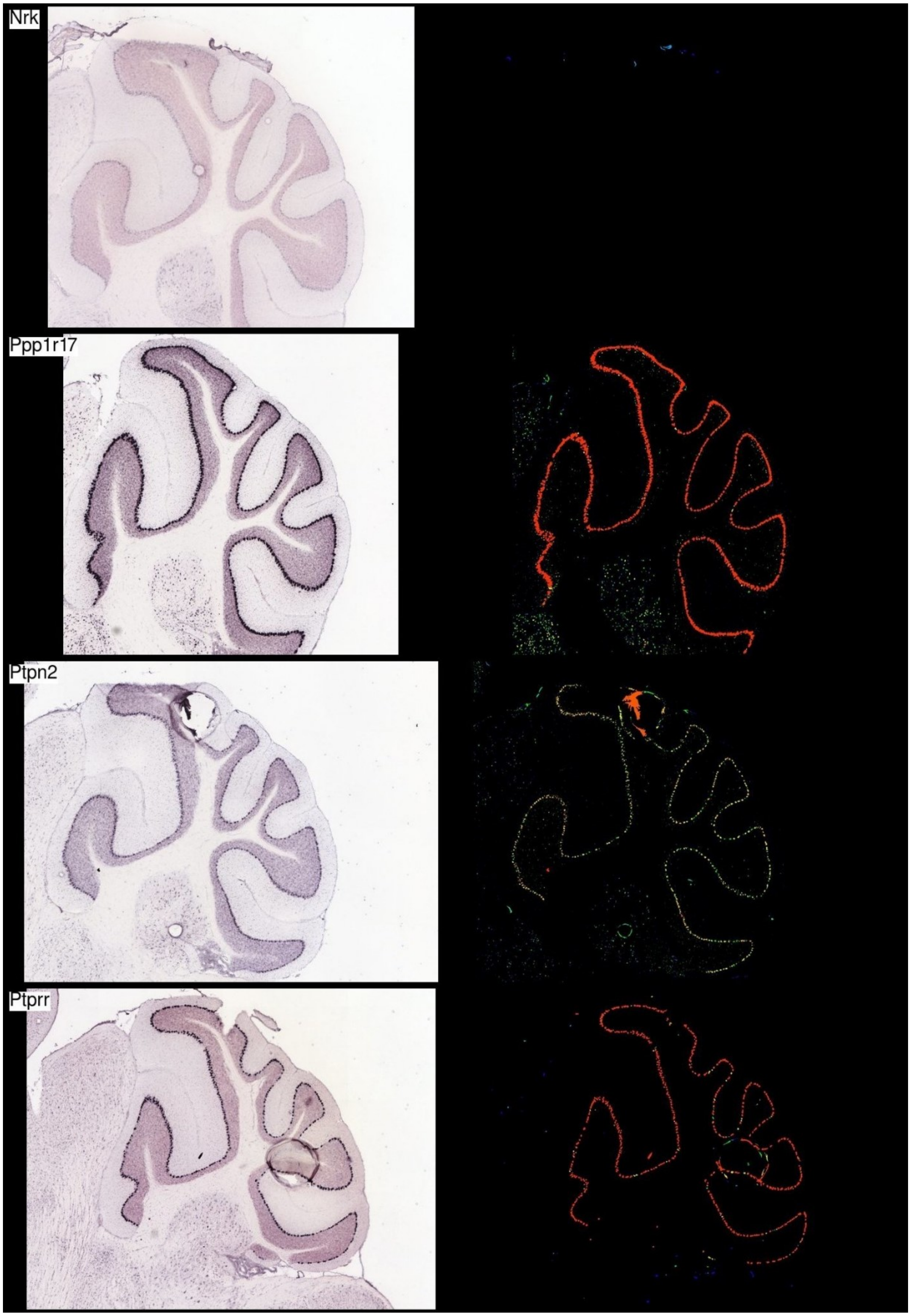


Figure 4-2 continued.

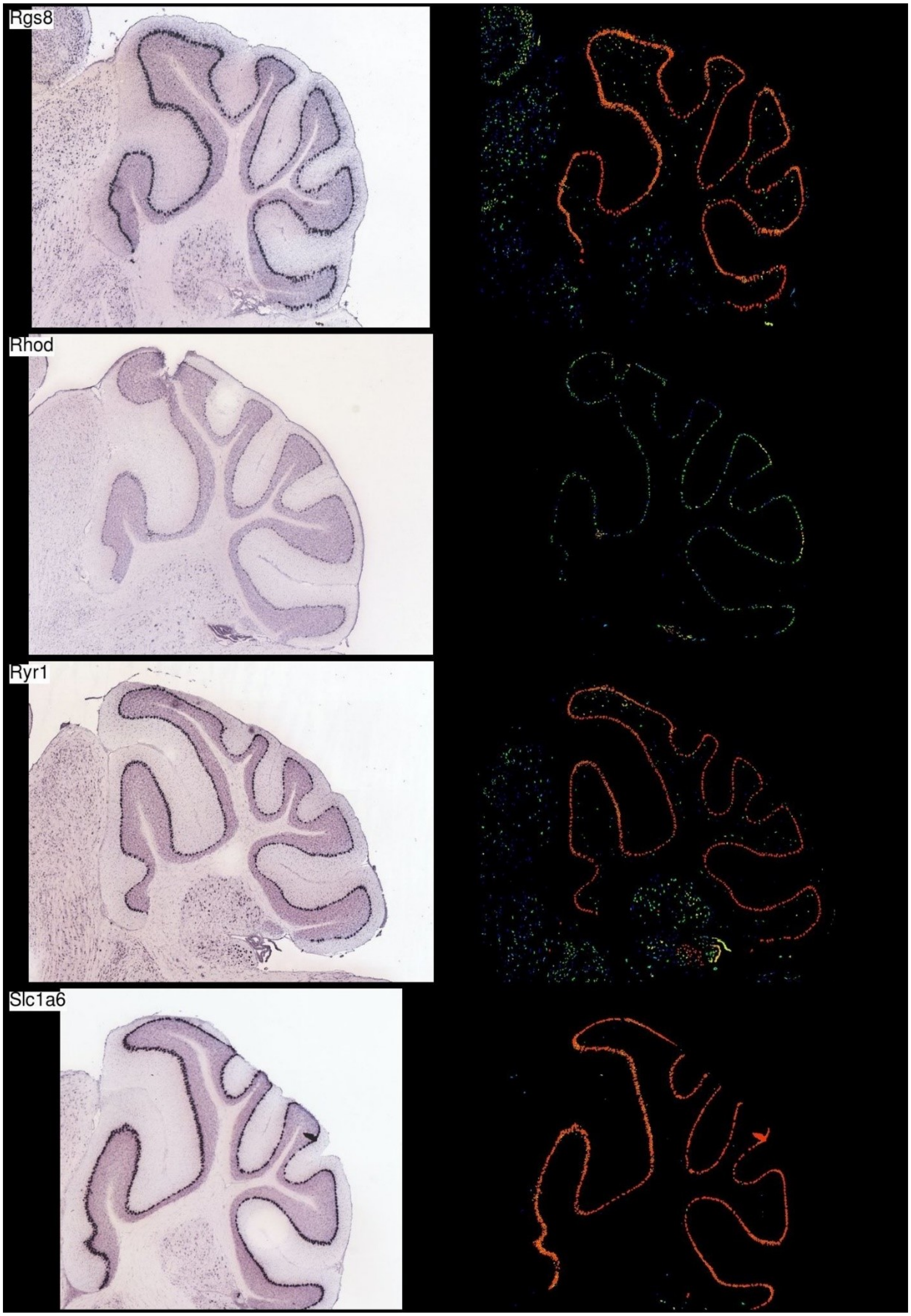


Figure 4-2 continued.

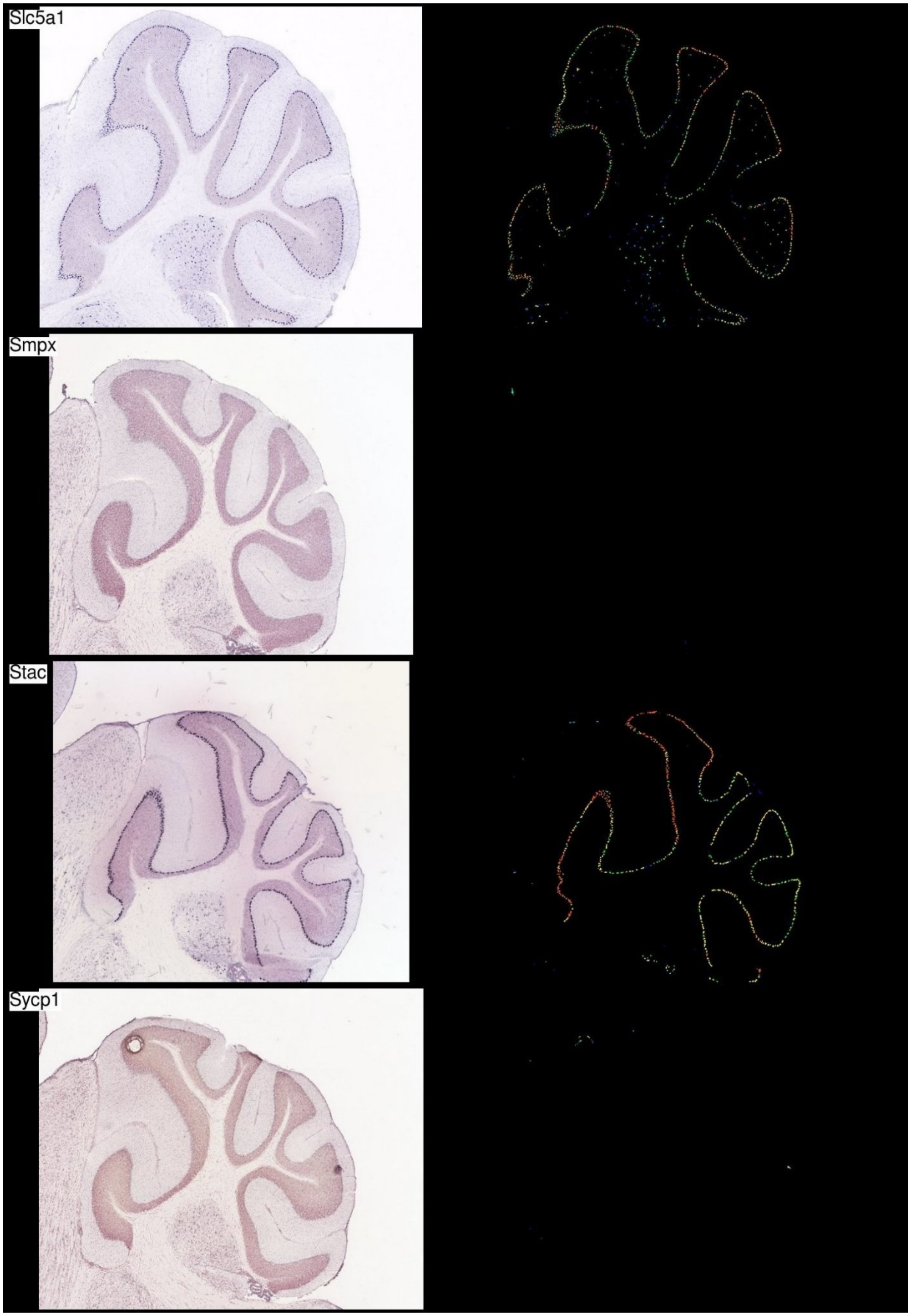


Figure 4-2 continued.

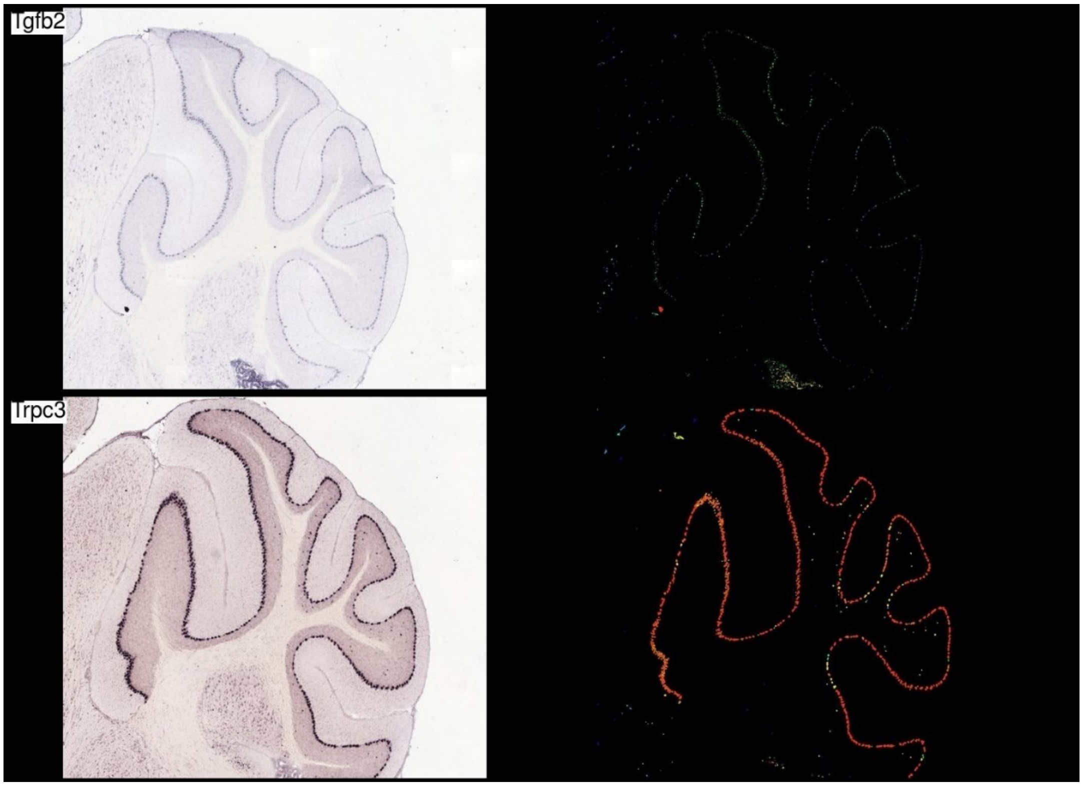


Figure 4-2 continued.

| Gene | Status | Notes |
|---|---|---|
| Cdhr1 | Ok | |
| Dsg2 | Ok | |
| Dsp | Ok | |
| Ehd1 | Ok | |
| Eml5 | Not specific | |
| Neurod1 | Ok | |
| Npnt | Ok | |
| Npy5r | Ok | |
| Ogn | Ok | Low expression |
| Pter | No signal | Not expressed anywhere else in the rest of the brain |
| Spint2 | Ok | |
| St8sia4 | Ok | Low expression |
| Stx3 | Ok | |
| Tdo2 | Ok | |
| Tiam1 | Ok | |
| Trpc6 | Ok | |

Figure 4-3: Validation status of dentate granule cell markers.

| Gene | Status | Notes |
|---|---|---|
| Atp2a3 | Ok | |
| B3gnt5 | Ok | |
| Bcl11a | Ok | 2nd probeset |
| Bean1 | Ok | |
| Cacna1g | Ok | |
| Cacna2d2 | Not specific | |
| Calb1 | Ok | |
| Casq2 | Ok | |
| Cck | Ok | |
| Col18a1 | Ok | |
| Dgkg | Ok | |
| Doc2b | Ok | |
| Ebf1 | Ok | |
| Ebf2 | Ok | Low signal |
| Eps8l2 | Inconclusive | |
| Fam174b | Not in ABA | |
| Gipc2 | Ok | Low signal |
| Gng13 | Ok | |
| Grik1 | Ok | |
| Homer3 | Ok | |
| Icmt | Ok | |
| Igfbp3 | Ok | Low signal |
| Igfbp6 | Ok | |
| Itpka | Ok | |
| Kcnip1 | Ok | |
| Krt25 | Ok | |
| Lhx5 | Ok | |
| Lmo7 | Ok | |
| Nrk | Ok | Low signal |
| Ppp1r17 | Ok | |
| Ptpn2 | Ok | |
| Ptprr | Ok | |
| Rgs8 | Ok | |
| Rhod | Ok | |
| Ryr1 | Ok | |
| Slc1a6 | Ok | |
| Slc5a1 | Ok | |
| Smpx | No signal | Not expressed anywhere else in the rest of the brain |
| Stac | Ok | |
| Sycp1 | No signal | Not expressed anywhere else in the rest of the brain |
| Tgfb2 | Ok | |
| Trpc3 | Ok | |
| Tuba8 | Not in ABA | |

Figure 4-4: Validation status of Purkinje cell markers.
