# Supplementary material for: Cross-Laboratory Analysis of Brain Cell Type Transcriptomes with Applications to Interpretation of Bulk Tissue Data
Source: eNeuro. 2017 Nov 30;4(6):ENEURO.0212-17.2017. doi: 10.1523/ENEURO.0212-17.2017 (PMC5707795; doi:10.1523/ENEURO.0212-17.2017)
Supplement: Extended Data 3 [file enu006172455so2.zip › neuroExpressoAnalysis-master/analysis/03.MarkerGeneProfiles/publishPlot/stanleyC.pdf]

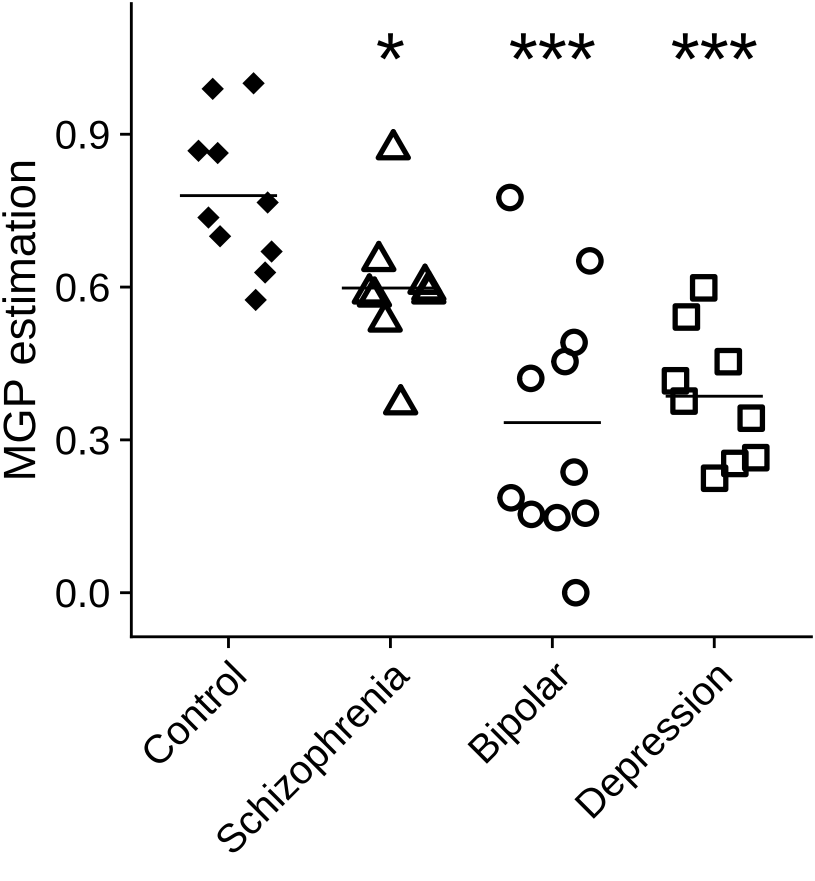
MGP estimation
0.9
0.6
0.3
0.0
*
***
***
Control
Schizophrenia
Bipolar
Depression
