# Supplementary material for: Cross-Laboratory Analysis of Brain Cell Type Transcriptomes with Applications to Interpretation of Bulk Tissue Data
Source: eNeuro. 2017 Nov 30;4(6):ENEURO.0212-17.2017. doi: 10.1523/ENEURO.0212-17.2017 (PMC5707795; doi:10.1523/ENEURO.0212-17.2017)
Supplement: Extended Data 3 [file enu006172455so2.zip › neuroExpressoAnalysis-master/analysis/04.ISHValidation/ISHsupplement.pdf]

Figure S1: Expression of dentate granule cell markers discovered in the study in Allen Brain Atlas mouse brain in situ hybridization database. The first gene is Prox1, a known marker of dentate granule cells. The intensity is color-coded to range from blue (low expression intensity), through green (medium intensity) to red (high intensity). All images except Ogn is taken from the sagittal view. Ogn is taken from the coronal view.

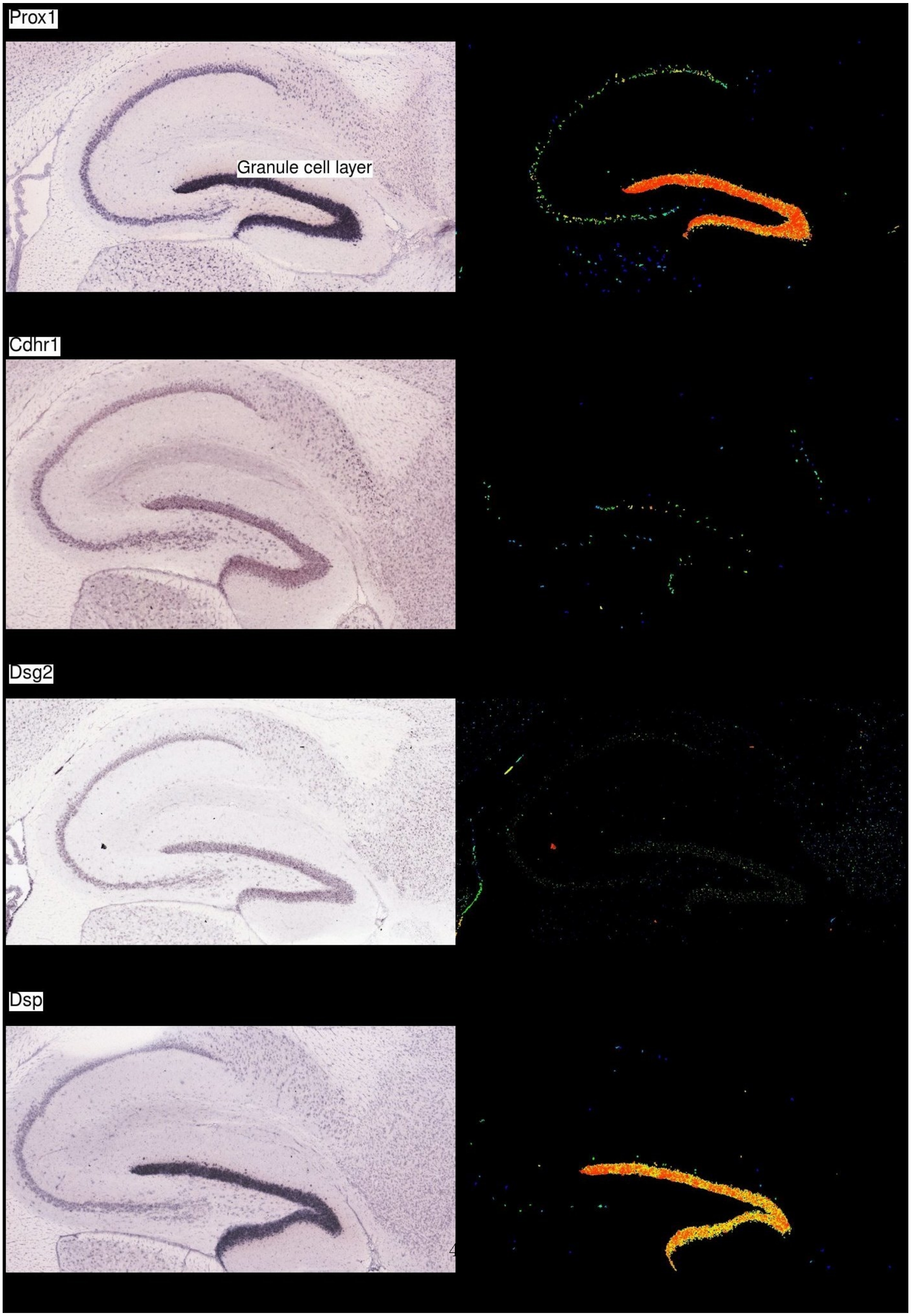

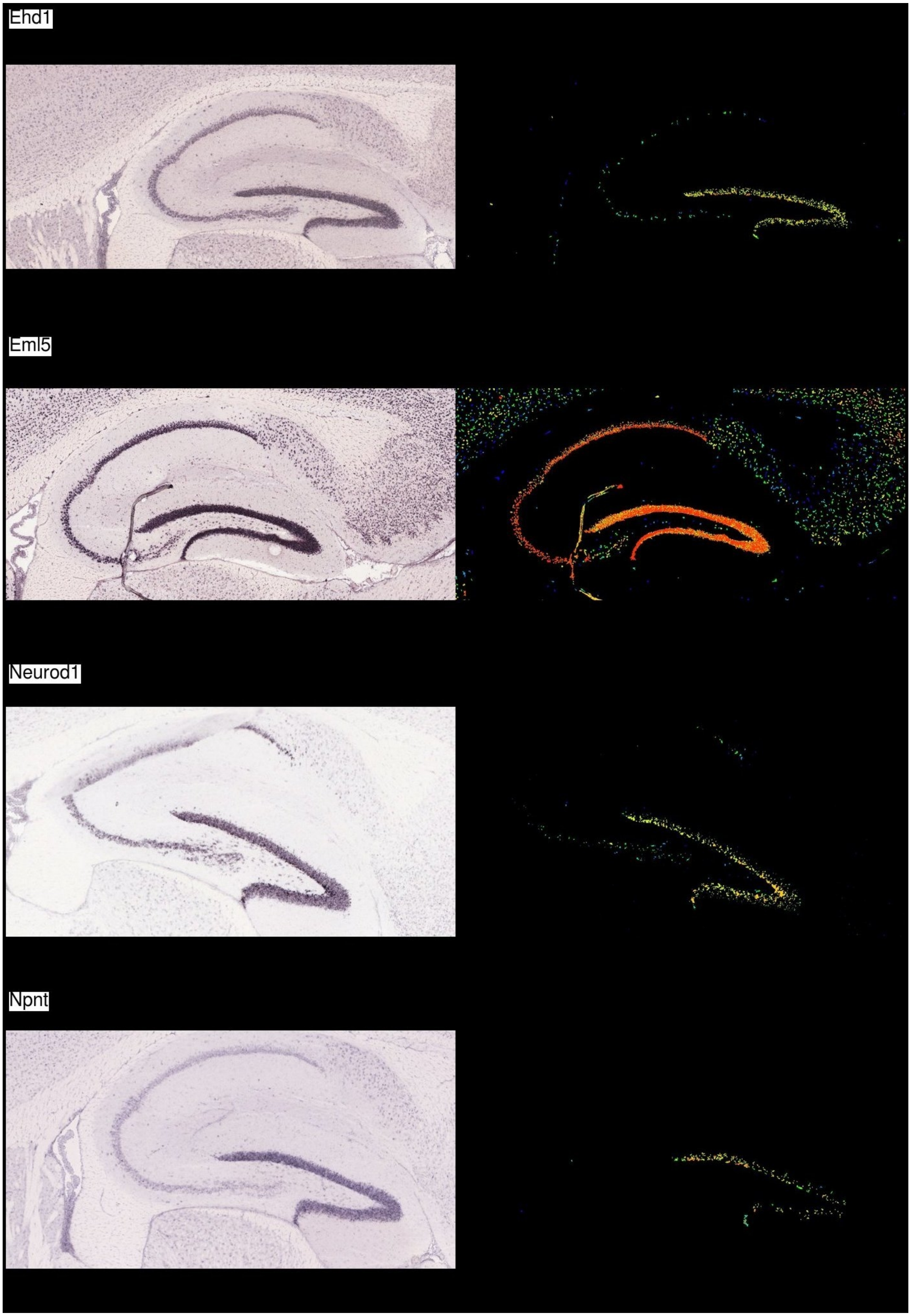
Ehd1
Eml5
Neurod1
Npnt

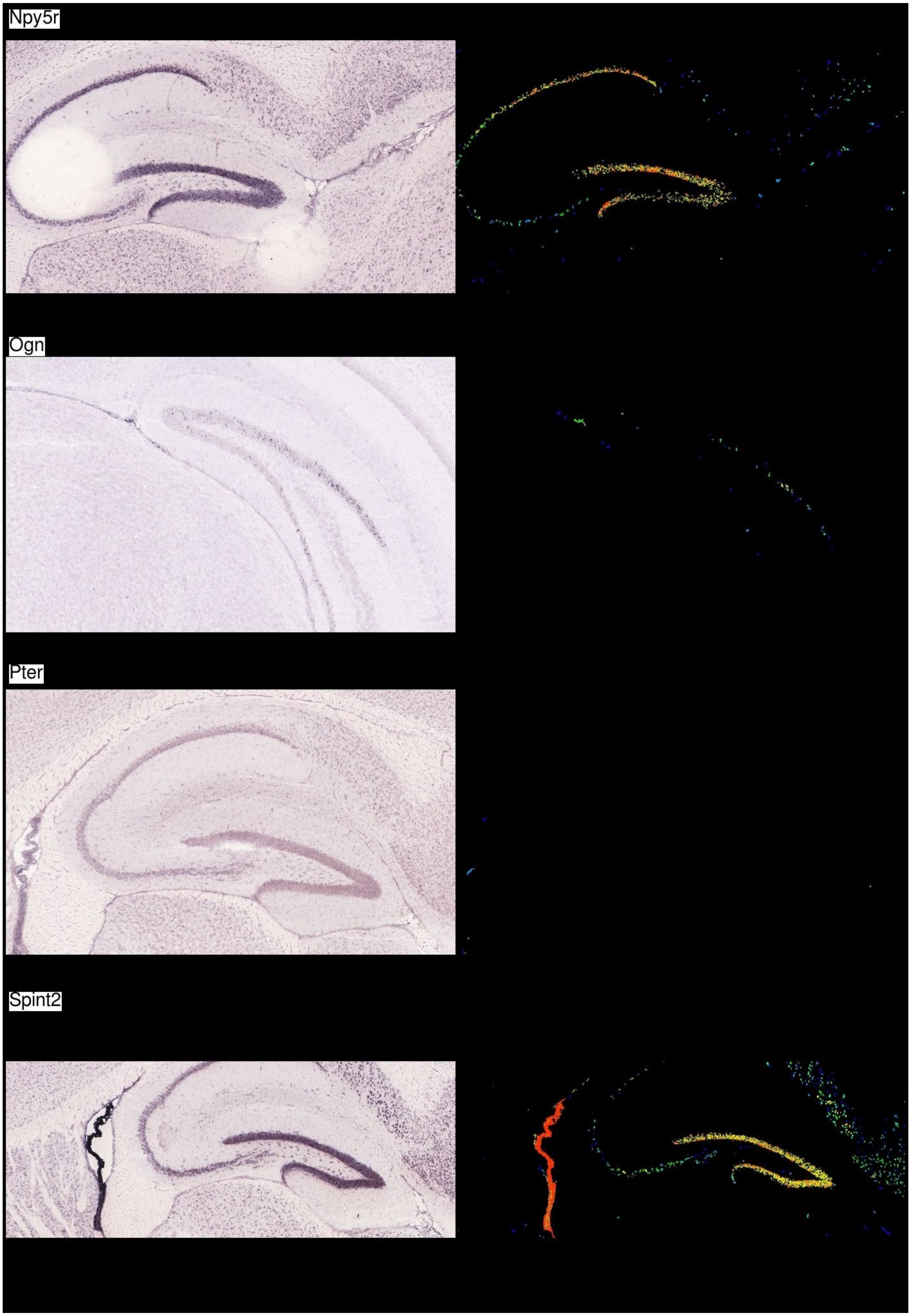
Npy5r
Ogn
Pter
Spint2

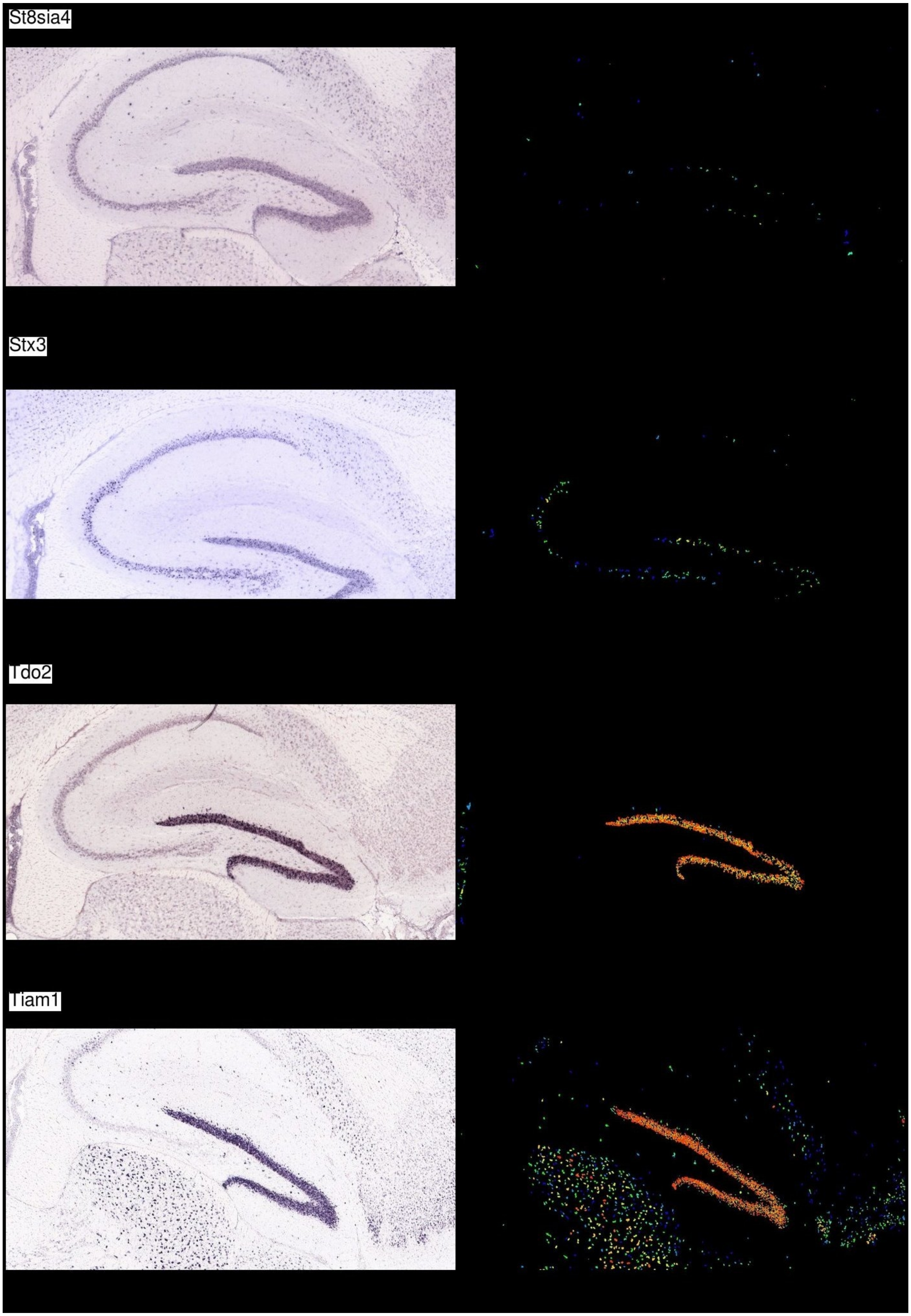
St8sia4
Stx3
Tdo2
Tiam1

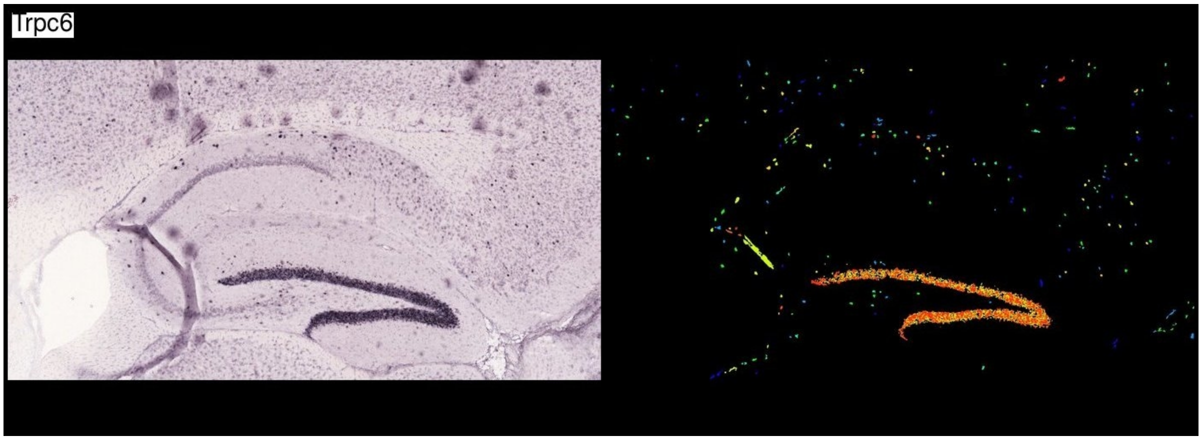
Trpc6

Figure S2: Expression of Purkinje markers discovered in the study in Allen Brain Atlas mouse brain in situ hybridization database. The first gene is Pcp2, a known marker of Purkinje cells. The intensity is color-coded to range from blue (low expression intensity), through green (medium intensity) to red (high intensity).

All images are taken from the sagittal view

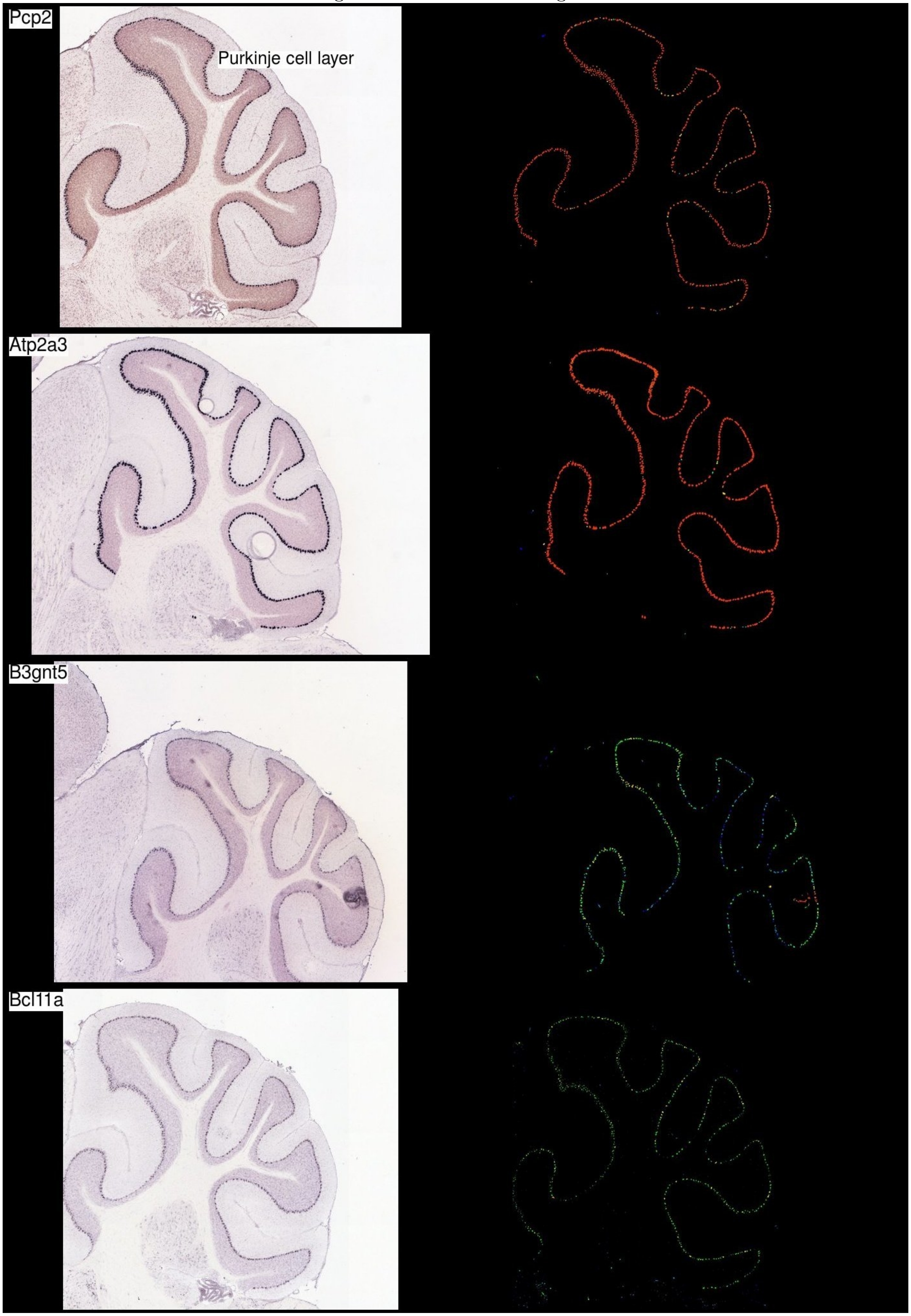

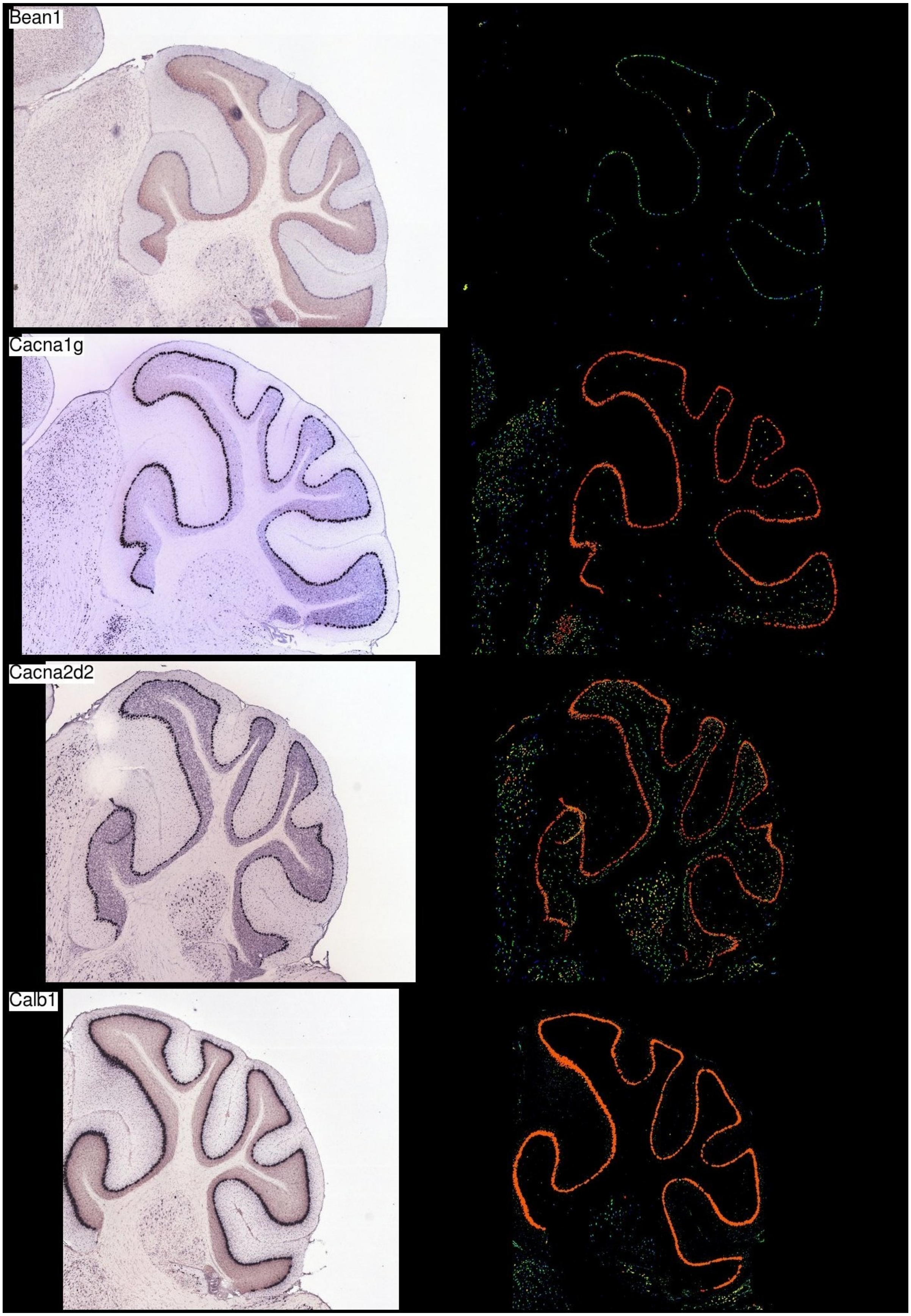
Bean1
Cacna1g
Cacna2d2
Calb1

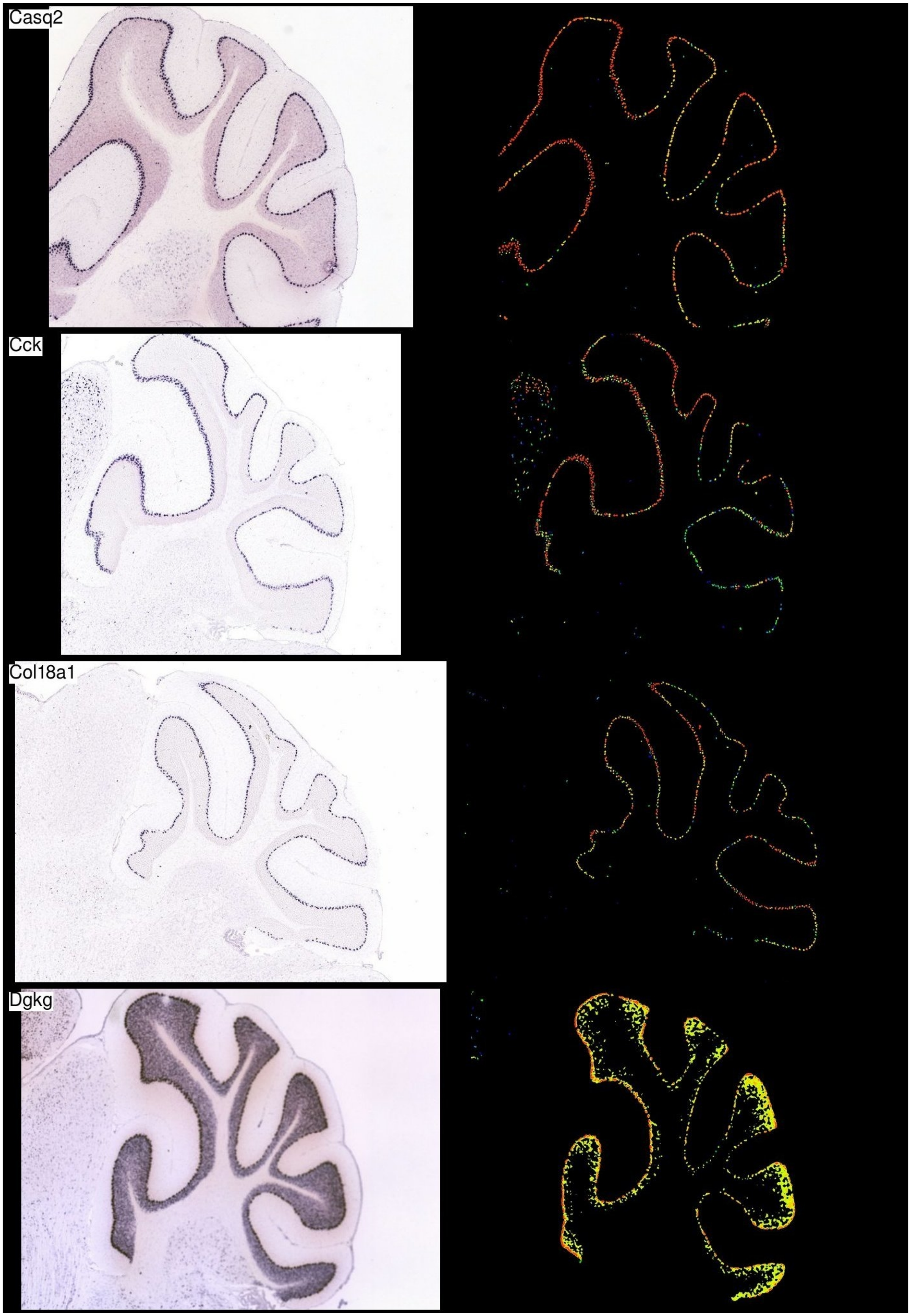
Casq2
Cck
Col18a1
Dgkg

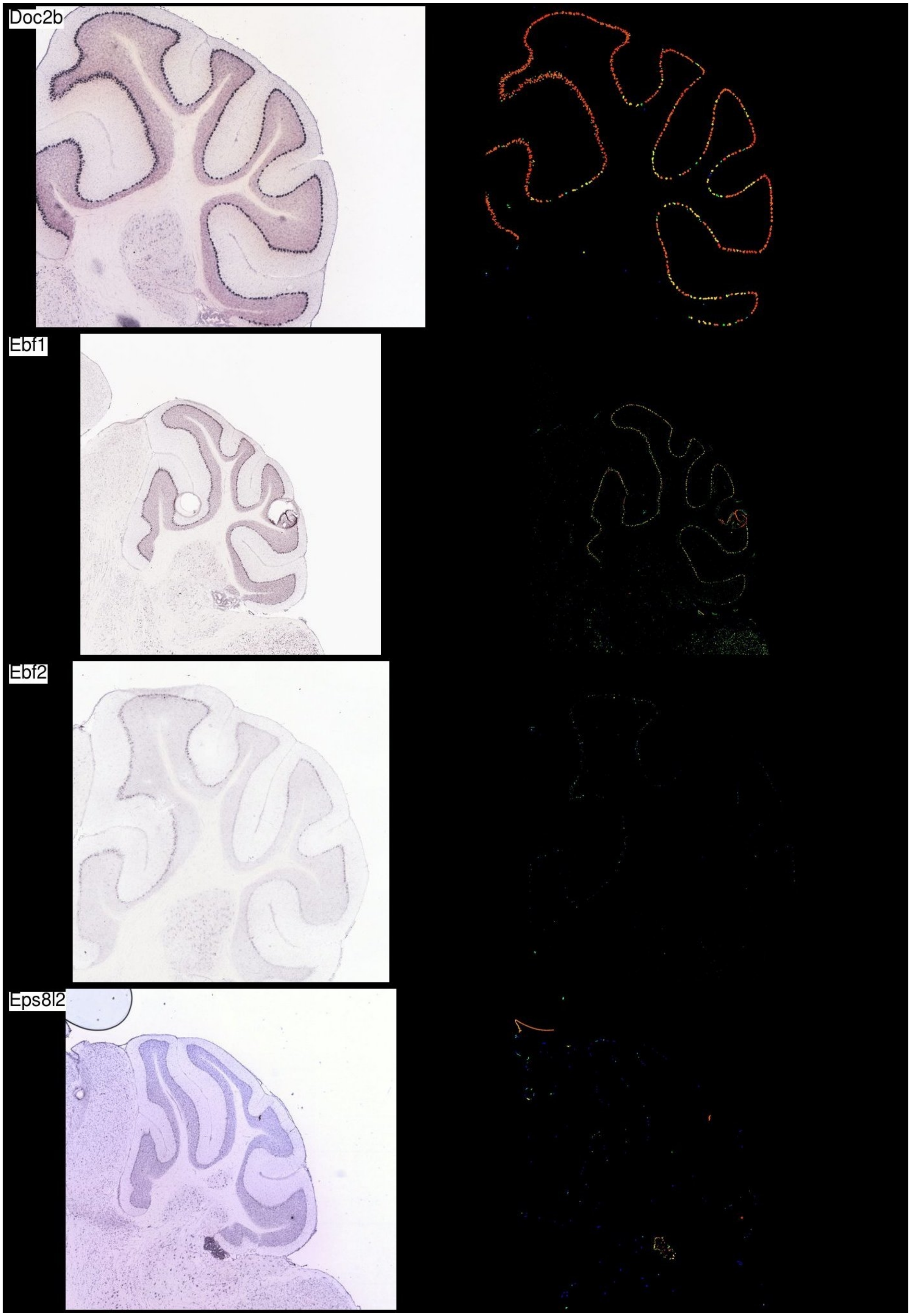

Doc2b
Ebf1
Ebf2
Eps8l2

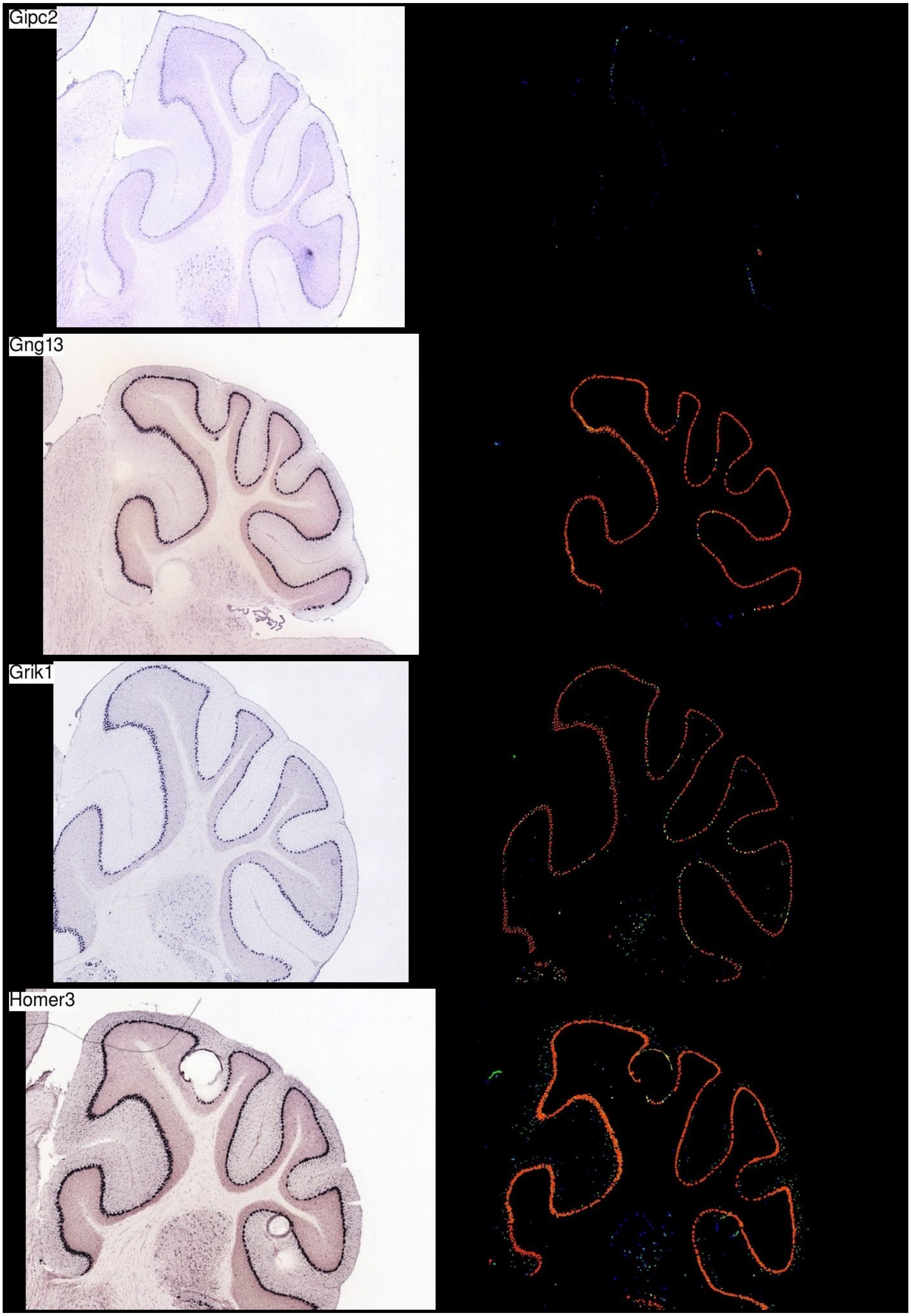
Gipc2
Gng13
Grik1
Homer3

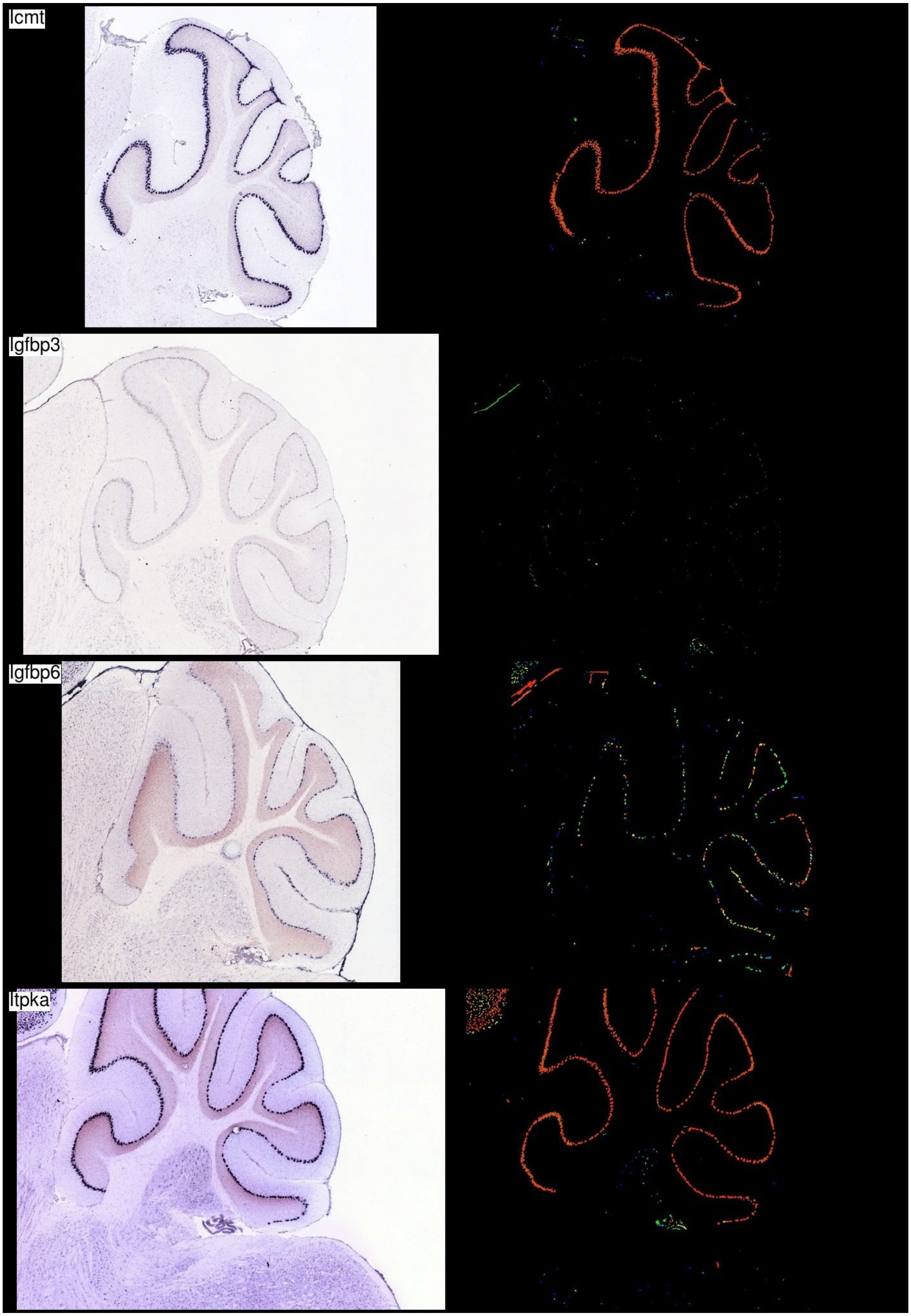
Icmt
Igfbp3
Igfbp6
Itpka

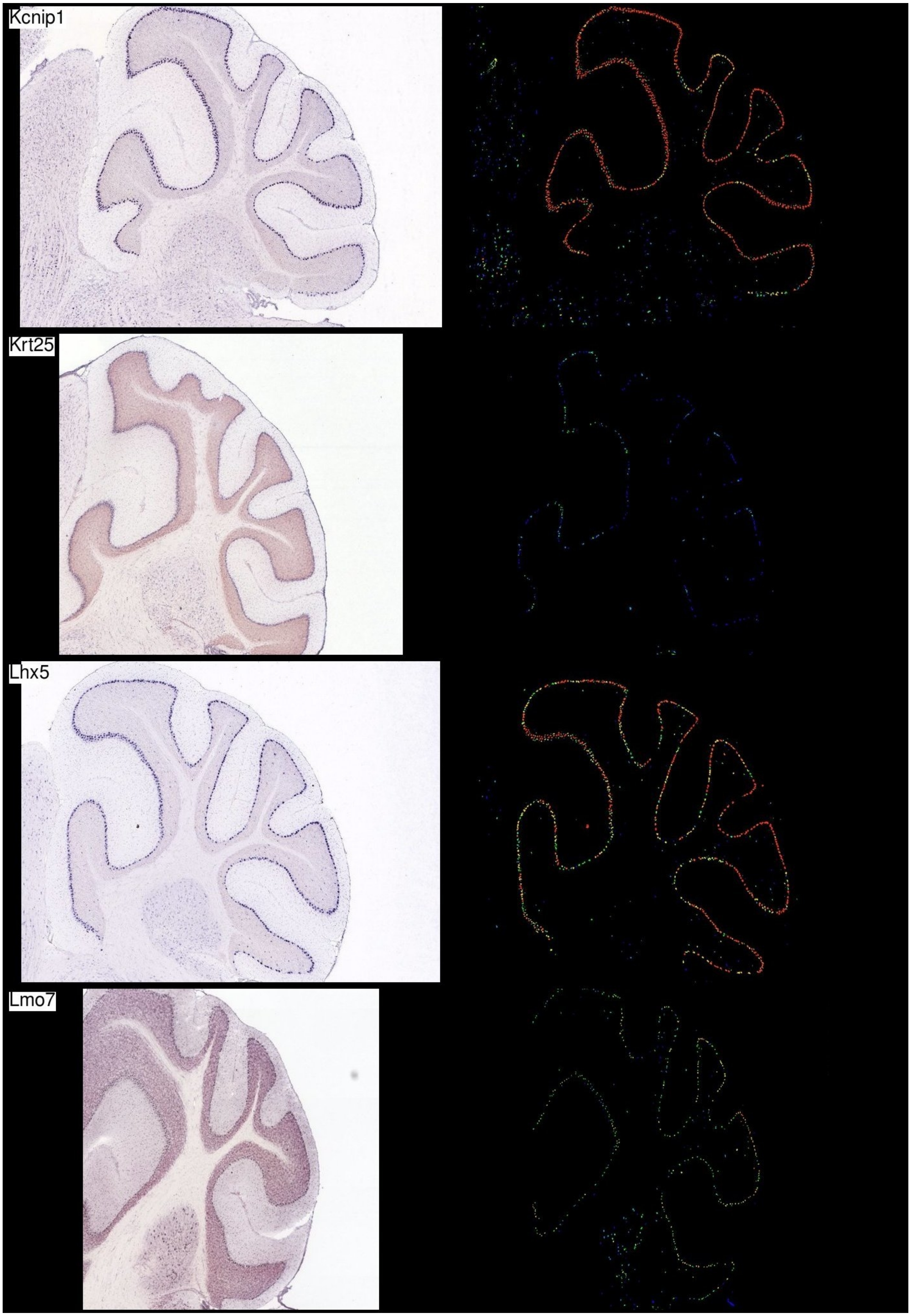
Kcnip1
Krt25
Lhx5
Lmo7

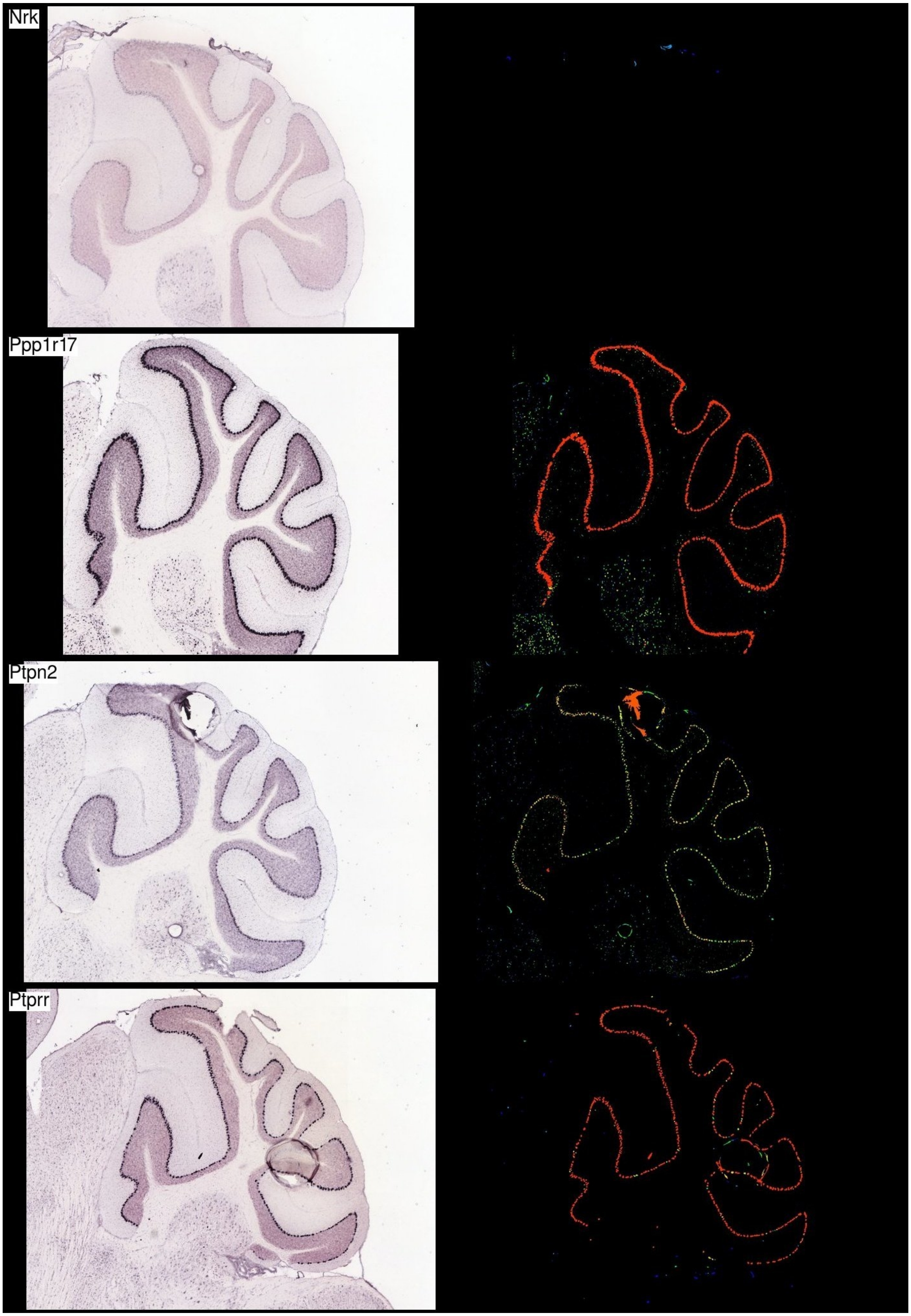
Nrk
Ppp1r17
Ptpn2
Ptprr

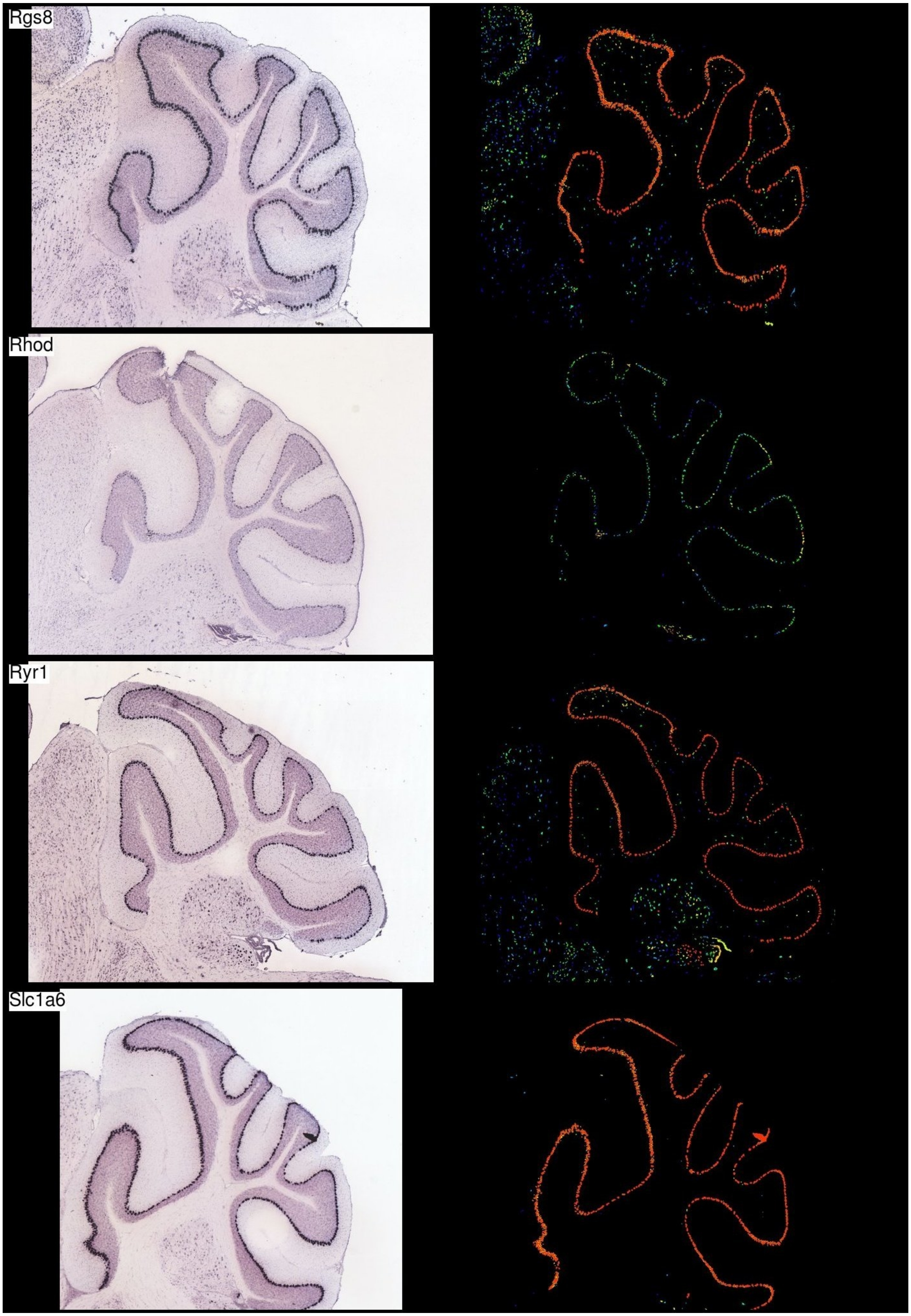
Rgs8
Rhod
Ryr1
Slc1a6

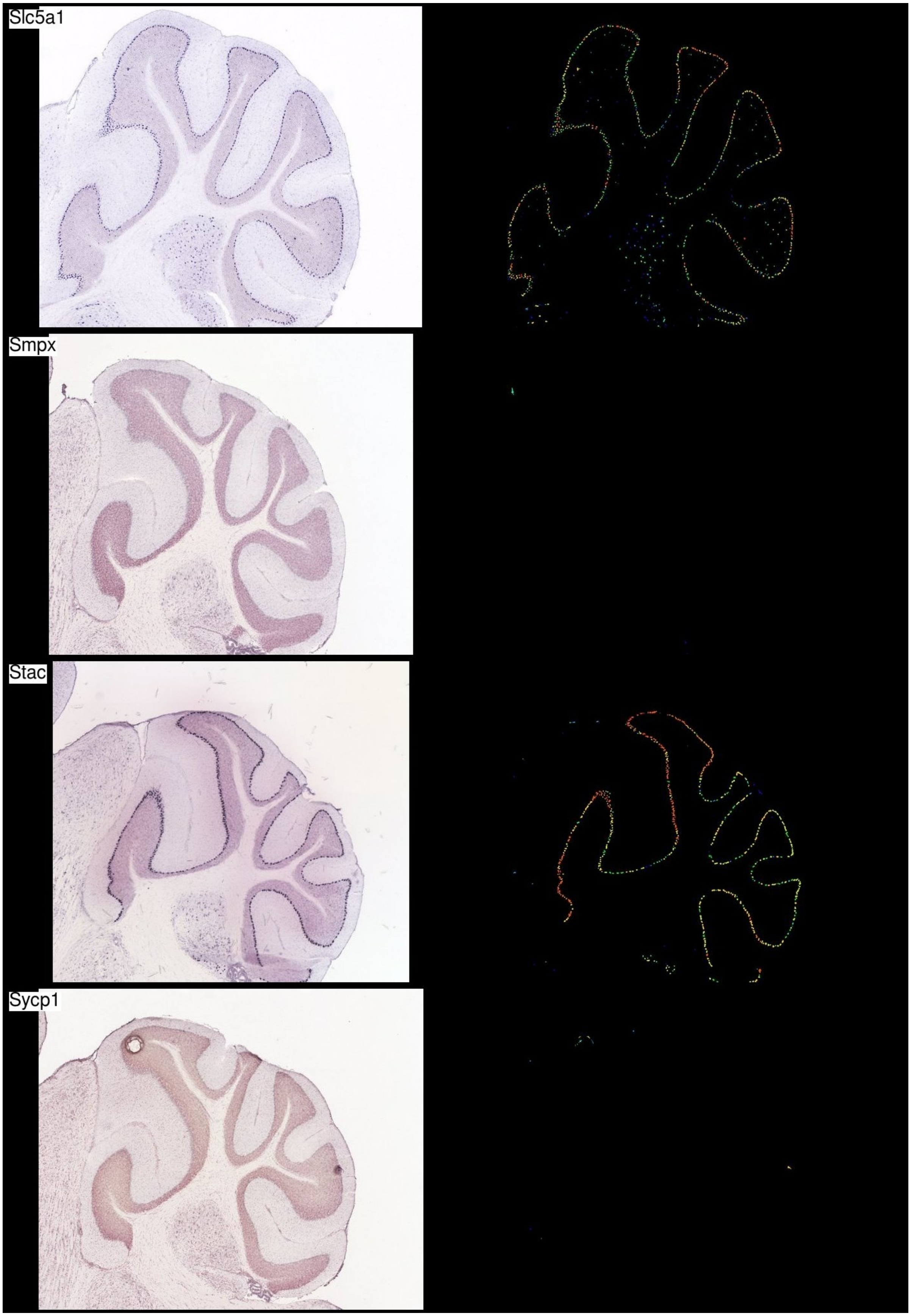
Slc5a1
Smpx
Stac
Sycp1

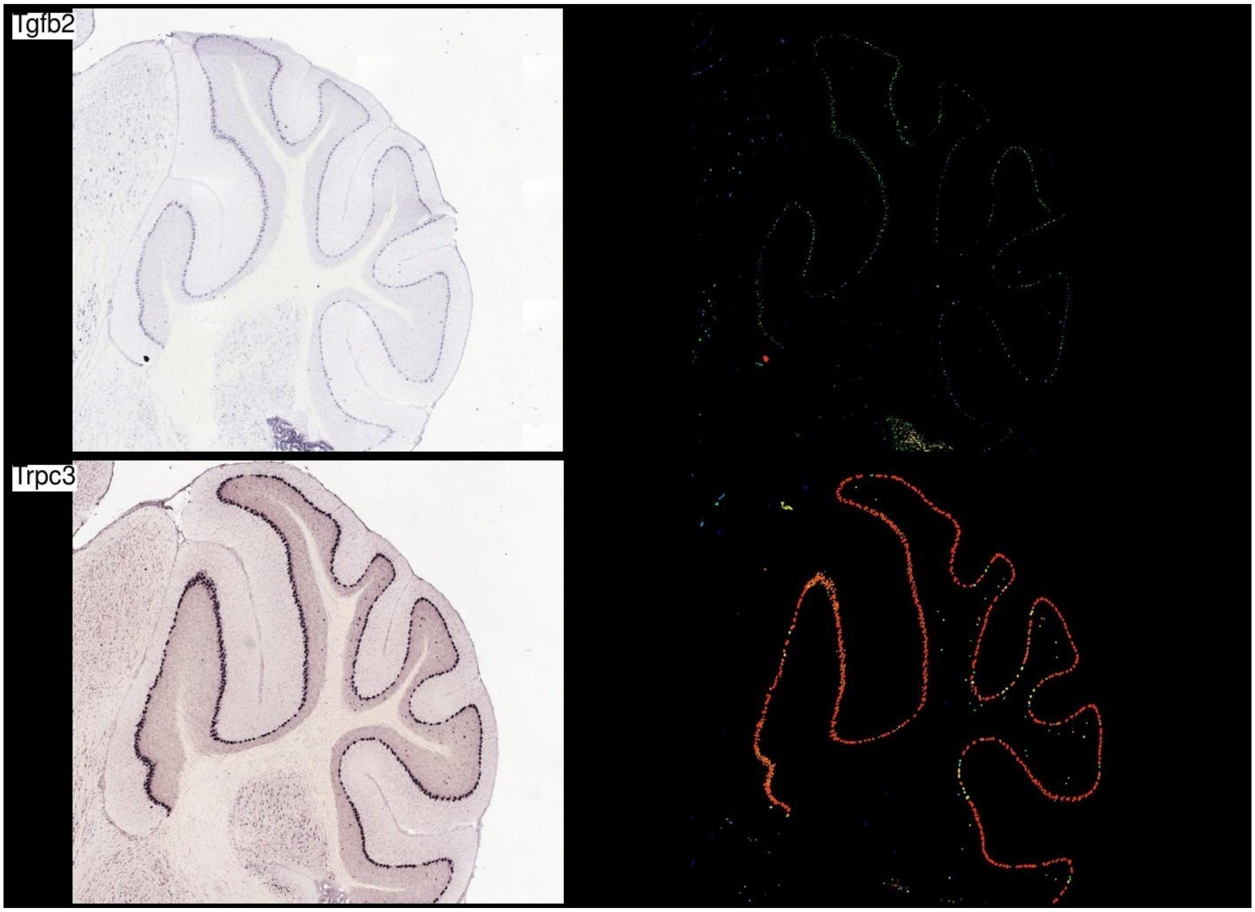

Table S1: Validation status of dentate granule cell markers.

Gene	Status	Notes
Cdhr1	ok	
Dsg2	ok	
Dsp	ok	
Ehd1	ok	
Eml5	not specific	
Neurod1	ok	
Npnt	ok	
Npy5r	ok	
Ogn	ok	low expression
Pter	not expressed	Not expressed anywhere else in the rest of the brain
Spint2	ok	
St8sia4	ok	low expression
Stx3	ok	
Tdo2	ok	
Tiam1	ok	
Trpc6	ok	

Table S2: Validation status of Purkinje cell markers.

Gene	Status	Notes
Atp2a3	ok	
B3gnt5	ok	
Bcl11a	ok	2nd probeset
Bean1	ok	
Cacna1g	ok	
Cacna2d2	not specific	
Calb1	ok	
Casq2	ok	
Cck	ok	
Col18a1	ok	
Dgkg	ok	
Doc2b	ok	
Ebf1	ok	
Ebf2	ok	Low signal
Eps8l2	inconclusive	
Fam174b	not in ABA	
Gipc2	ok	Low signal
Gng13	ok	
Grik1	ok	
Homer3	ok	
Icmt	ok	
Igfbp3	ok	Low signal
Igfbp6	ok	
Itpka	ok	
Kcnip1	ok	
Krt25	ok	
Lhx5	ok	
Lmo7	ok	
Nrk	ok	Low signal
Ppp1r17	ok	
Ptpn2	ok	
Ptprr	ok	
Rgs8	ok	
Rhod	ok	
Ryr1	ok	
Slc1a6	ok	
Slc5a1	ok	
Smpx	not expressed	Not expressed anywhere else in the rest of the brain
Stac	ok	
Sycp1	not expressed	Not expressed anywhere else in the rest of the brain
Tgfb2	ok	
Trpc3	ok	
Tuba8	not in ABA	

Table S3: Intersection of Purkinje markers from NeuroExpresso markers and Rong et al. 2004 study. Rong et al. genes are taken from Table 2 of the paper. Probe to gene annotations are repeated using annotations from Gemma. Fold change column shows the difference of expession between wild type and pcd^{3J} mouse which lacks Purkinje cells according to Rong et al.

Gene Symbol	Probeset	Fold change
Slc1a6	1418933_at	2.03
Cck	1419473_a_at	3.53
Gng13	1419414_at	3.88
Ppp1r17	1449240_at	4.12
Calb1	1417504_at	5.63
Calb1	1448738_at	7.42
Itpka	1424037_at	7.76
Cacna1g	1423365_at	12.51
Doc2b	1420667_at	14.08
Icmt	1426500_at	14.11
Rgs8	1453060_at	15.17
Nrk	1450079_at	15.83
Casq2	1422529_s_at	16.36
Trpc3	1417577_at	21.03
Kcnip1	1448459_at	21.41
Homer3	1424859_at	27.80
Atp2a3	1421129_a_at	28.17
Kcnip1	1416785_at	28.50
Atp2a3	1450124_a_at	31.57
Sycp1	1427291_at	33.16
Doc2b	1420666_at	36.04
Ptprr	1426047_a_at	36.41
Fam174b	1434273_at	43.73
Slc5a1	1419057_at	46.80
